# iGenomics: Comprehensive DNA sequence analysis on your Smartphone

**DOI:** 10.1093/gigascience/giaa138

**Published:** 2020-12-07

**Authors:** Aspyn Palatnick, Bin Zhou, Elodie Ghedin, Michael C Schatz

**Affiliations:** Cold Spring Harbor High School, 82 Turkey Lane, Cold Spring Harbor, NY 11724, USA; Simons Center for Quantitative Biology, Cold Spring Harbor Laboratory, One Bungtown Road, Cold Spring Harbor, NY 11724, USA; Networked and Social Systems Engineering, University of Pennsylvania, 220 South 33rd Street, Philadelphia, PA 19104, USA; Department of Biology, New York University, 100 Washington Square, New York, NY 10003, USA; Department of Biology, New York University, 100 Washington Square, New York, NY 10003, USA; Department of Epidemiology, New York University School of Global Public Health, 665 Broadway St, New York, NY 10003, USA; Simons Center for Quantitative Biology, Cold Spring Harbor Laboratory, One Bungtown Road, Cold Spring Harbor, NY 11724, USA; Departments of Computer Science and Biology, Johns Hopkins University, 3400 N Charles St, Baltimore, MD 21211, USA

**Keywords:** DNA sequencing, variant analysis, mobile computing

## Abstract

**Background:**

Following the miniaturization of integrated circuitry and other computer hardware over the past several decades, DNA sequencing is on a similar path. Leading this trend is the Oxford Nanopore sequencing platform, which currently offers the hand-held MinION instrument and even smaller instruments on the horizon. This technology has been used in several important applications, including the analysis of genomes of major pathogens in remote stations around the world. However, despite the simplicity of the sequencer, an equally simple and portable analysis platform is not yet available.

**Results:**

iGenomics is the first comprehensive mobile genome analysis application, with capabilities to align reads, call variants, and visualize the results entirely on an iOS device. Implemented in Objective-C using the FM-index, banded dynamic programming, and other high-performance bioinformatics techniques, iGenomics is optimized to run in a mobile environment. We benchmark iGenomics using a variety of real and simulated Nanopore sequencing datasets of viral and bacterial genomes and show that iGenomics has performance comparable to the popular BWA-MEM/SAMtools/IGV suite, without necessitating a laptop or server cluster.

**Conclusions:**

iGenomics is available open source (https://github.com/stuckinaboot/iGenomics) and for free on Apple's App Store (https://apple.co/2HCplzr).

## Background

DNA sequencing technology has made tremendous progress over the past 30 years [1]. The earliest automated approaches, beginning with the capillary-based Sanger sequencing devices in the 1980s,  were large bench-top instruments requiring extensive sequencing facilities to prepare and sequence the DNA. In the 2000s,  high-throughput second-generation sequencing instruments advanced the field with more compact and simpler designs. However, these advances have been limited in their reach because they are not readily accessible by most individual laboratories and citizen scientists. Most substantially, the most widely used alignment and analysis tools are not targeting citizen scientists and require expert knowledge on using the command line to install several software packages, run the tools, and understand a variety of file formats.

Within the past few years, Oxford Nanopore Technologies (ONT, Oxford, UK) has introduced a small inexpensive hand-held sequencing instrument that has made it possible to perform genomics experiments with minimal facilities and in essentially any environment. Because of its small size, Nanopore sequencing has been used in several environments that would be unthinkable for alternative instruments as diverse as monitoring the Ebola outbreaks in remote areas of Africa [2], monitoring Zika outbreaks in South America [3], exploring reptile specimens in the rainforest [4], and even on the International Space Station [5]. Nanopore sequencing has also played an important role in monitoring the transmission of SARS-COVID-19 around the world [6–8]. Nanopore sequencing technology works by measuring the change in ionic current as a DNA molecule is passed through a nanopore [1]. The DNA molecules are typically a few hundred to tens of thousands of nucleotides long, and the longest reported read has exceeded 2 million nucleotides [9]. Once sequenced, the raw signal data are base-called into nucleotide strings called reads [10], which are typically stored in fastq format and saved for further processing, especially read alignment and variant analysis.

Several algorithms are available for this analysis. Modern aligners, such as Bowtie [11] or BWA-MEM [12], often use the Burrows-Wheeler transform (BWT) [13] and the closely related FM-index [14] as their core indexing data structure. These new approaches are suited to large datasets because of their compact space requirements and fast alignment times. After alignment, variant-calling platforms, such as SAMtools [15] or GATK [16], systematically scan the alignments to find well-supported variants in the sample using a statistical model to distinguish homozygous from heterozygous variants and rule out spurious sequencing errors. After this automated variant identification, high-priority variants are also often manually inspected using IGV [17] and other genome browsers to review the evidence for the variant calls and further rule out false-positive calls.

The standard approach for analyzing reads is to align the reads to a reference genome on high-end laptops, servers, or even supercomputers. While this is feasible for those with access to these technologies, these requirements may be out of reach for many researchers and citizen scientists. Instead, iGenomics just requires the sequenced reads, which can be loaded from the phone itself, the internet, or elsewhere, and can allow anyone to perform sequence analysis and mutation identification. As with other mobile applications (e.g., web browsing, e-mail, social media), iGenomics can be used in a variety of settings where it would be awkward to use a larger laptop, and many users will also prefer the more intuitive user interface. Furthermore, there are many important scenarios where analyzing these data without high-end computing hardware is desirable, especially in remote environments. Interestingly, current iOS devices, including both iPads and iPhones, have significant computing resources, with clock speeds and onboard RAM approaching that of high-end laptop computers. That said, no stand-alone genomics analysis software is currently available for iOS devices.

Addressing this critical gap, we have developed iGenomics, an iOS application that allows anyone to easily align and analyze DNA sequences in a mobile environment. iGenomics uses the same high-performance algorithms for read alignment and variant calling as mainstream software, although iGenomics marks the first time these algorithms have been implemented in a mobile iOS environment. Additionally, using the advanced user interface features available in iOS, iGenomics allows for interactive visualization and inspection of the read alignments and variant calls, and contains additional features for reviewing critical mutations of interest. For example, iGenomics comes bundled with a listing of critical mutations in the influenza A virus that indicate which antiviral agents are most likely to be ineffective [18].

Owing to the lower amount of processing power in mobile devices compared with high-end desktop computers or servers, iGenomics is limited in the size of the genome that can be processed. However, the implementations in iGenomics have been rigorously tested through direct comparisons with the BWA-MEM/SAMtools framework for alignment and variant calling for viral and microbial genomes. All alignment and analysis algorithms used by iGenomics have been tested on both real and simulated datasets to ensure consistent speed, accuracy, and reliability of both alignments and variant calls. Consequently, iGenomics is leading the shift of DNA analysis software and sequencing tools towards mobile devices and marks a great leap forward towards widespread DNA analysis by non-bioinformatician students, researchers, and citizen scientists. Furthermore, iGenomics is available open source to facilitate mobile genomics technology research and, in turn, accelerate the speed at which this technology is developed.

## Results

### Interactive sequence analysis on your smartphone

**Figure 1:**
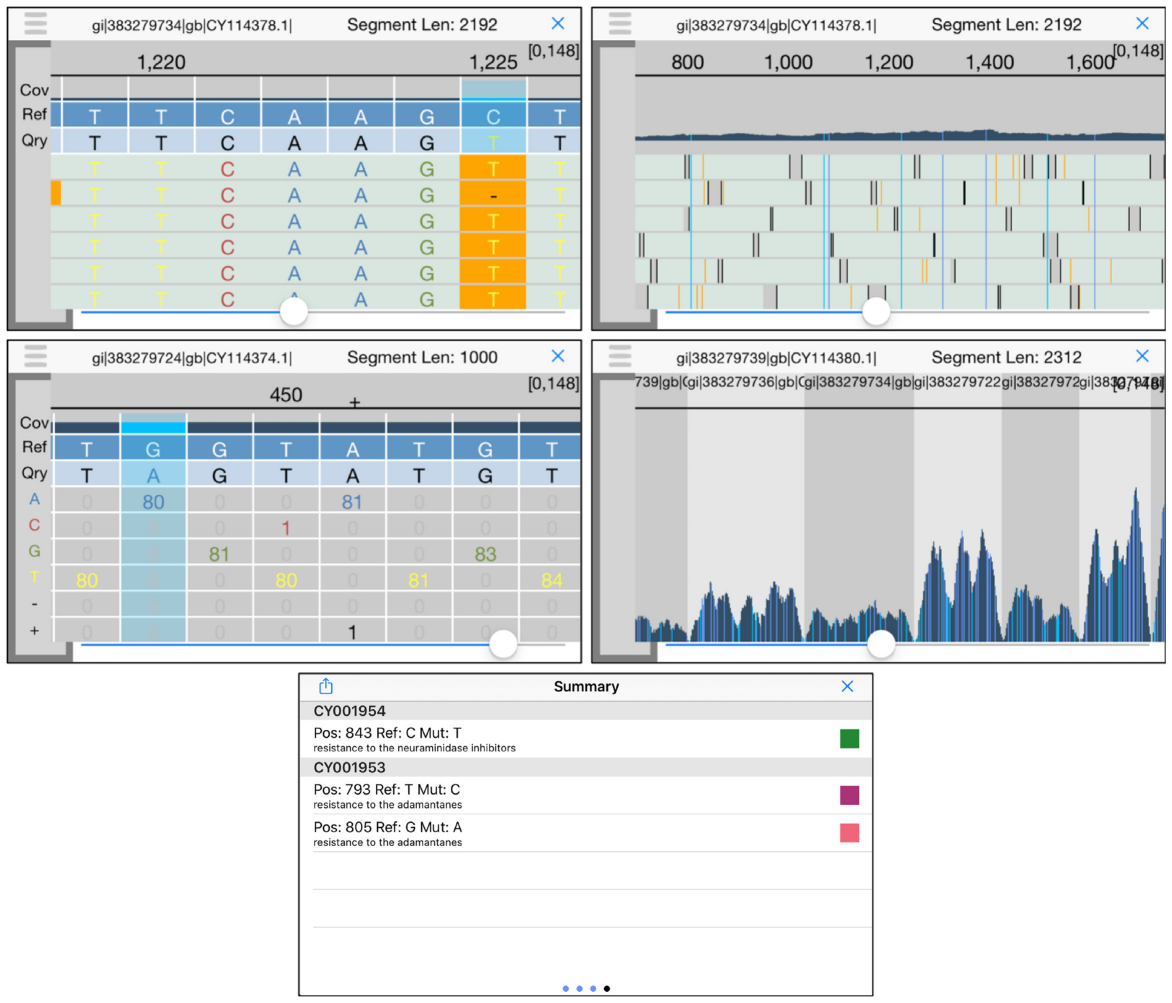
iGenomics iPhone screenshots (top left) alignments display, (top right) alignment display zoomed out, (middle left) coverage profile, (middle right) coverage profile zoomed out, and (bottom) known mutations display. In the known mutations display, green indicates the mutation is not present, purple indicates the listed mutation is present and the mutation is homozygous, and pink indicates the listed mutation is present and the mutation is heterozygous. In both the alignments display and coverage profile, there is an indicator in the top right of the form [X, Y] that represents the minimum coverage X across all positions and maximum coverage Y across all positions.

iGenomics brings a high level of interaction to DNA sequence analysis (Fig. 1). Common touchscreen gestures allow for users to browse the alignment data in an easy-to-use and intuitive manner. This allows the app to be used with almost no learning curve.

The first step of analysis is selecting the reads and a reference genome for analysis in either fasta or fastq format. iGenomics provides multiple options for inputting both reads and reference files: selecting from a variety of default files for common bacterial genomes, using Dropbox to choose a file, or loading a fasta or fastq file straight into iGenomics from another app such as Google Drive, Files, or Airdrop. Then, from a single view, the user can choose the reads file, the reference file, and, optionally, a tab-delimited file annotating known important mutations. For example, iGenomics comes with a preloaded known mutations file that indicates certain mutations in the influenza genome, which, if present, cause resistance to certain antiviral agents [18]. This single-view design is meant to be simple and requires minimal user effort. After choosing the files to align, the user can either select the “Analyze” button to align reads to the reference genome using the default parameters or choose to configure certain parameters before aligning. The parameters available include the maximum error rate for alignments and enabling trimming for fastq files.

After alignment completes, the user is brought to the analysis pane. The main view, known as the alignments display, is an IGV-like rendering of how the reads are aligned to a reference genome, with the ability to scroll left, right, up, and down through all of the aligned reads. Aligned bases that differ from the reference base are highlighted in a different color, as are consensus calls. A long touch on a read presents additional details about the read, including the read name, the edit distance of the alignment, the gapped read and gapped substring of the reference genome that the read aligned to, and whether the forward read or the reverse complement aligned. The user can also use the pinch gesture to zoom out, revealing a high-level overview of the individual alignments as well as a coverage profile of the number of reads that aligned at each position. Mutations are still highlighted after zooming out, allowing the user to see where all of the mutations occur in 1 view.

Another view within the analysis pane is the coverage profile, which displays the count of each base that aligned at each position. Positions where the reference base does not match the base of the reads are highlighted so that the user can see that this position contains a mutation (heterozygous mutations are highlighted with a different color). To scroll through the coverage profile, the user simply has to swipe left or right. If a user would like to view more detailed information about a given position, he/she simply holds down any of the boxes in that position and an informative view elaborating upon the position's contents will pop up. By using the pinch gesture to zoom out, the user reveals a graph of the number of reads that aligned at each position, resembling that of the zoomed-out alignments display but with a full-screen graph.

The Summary window, accessible from within the analysis pane, has 4 pages and provides some useful tools for a high-level overview of the data. The first page provides buttons to view the alignments display, coverage profile, coverage histogram, and list of all found mutations. The coverage histogram graphs the frequency of each level of coverage, specifically the frequency of a particular number of reads aligned to a position, and is overlaid by a Poisson curve for context. Within the list of all found mutations, the user can scroll through all mutations and then select one to inspect that position in the analysis pane. The second page gives an overview of the alignments, including the percentage of reads matched, the total number of reads input, the number of mutations, and the names of the reads and reference files. On this page the user can also search for positions in the reference genome by position or by a query string, which uses BWT exact match for rapid searching. The third page contains a large picker view that allows the user to intuitively move between sequences/segments in the reference genome. The last page contains a list of known mutations if the user selected a known mutations file during the file input stage. This list contains mutation position, mutation details (such as resistance to antivirals), and a color-coded indicator denoting whether a mutation was found at that position and whether that mutation indicates a known mutation.

### Simulated read runtime analysis

To observe the efficiency and accuracy of iGenomics running on an iPhone 8, we first tested several simulated datasets. The reference genomes we used were as follows:

1. phiX174, a widely used control sequence for Illumina sequencing (Genbank: NC_001422.1, 5,386 bp);
2. a Zika virus genome (isolate Zika virus/H.sapiens-tc/KHM/2010/FSS13025, 10,807 bp);
3. an H3N2 influenza genome (A/California/7/2004(H3N2), 13,382 bp);
4. an H1N1 influenza genome (A/New York/205/2001(H1N1), 13,568 bp); and
5. an Ebola genome (isolate Ebola virus/H.sapiens-wt/SLE/2014/Makona-G3686.1, 18,957 bp).

From these reference genomes, we then simulated reads using DWGSIM [19] according to the following conditions: the mean coverage is 100×, the genetic mutation rate was set to 0.5%, and the read characteristics would mirror reads produced by real-world sequencers. Accordingly, reads of length 100 bp and sequence error rate of 1.0% were simulated to mirror reads generated by Illumina sequencers and reads of length 1,000 bp and sequence error rate of 10.0% were simulated to mirror reads generated by ONT sequencers. Sequencing errors were introduced at random to mimic the errors produced by sequencers. For comparison purposes, we also measured the runtime when aligning and identifying variations using BWA-MEM [12] using “-x ont2d” and SAMtools pipeline for the same datasets. Notably, iGenomics uses an FM-index and banded dynamic programming implementation similar to BWA-MEM, allowing the analysis to focus on major differences in hardware.

**Figure 2:**
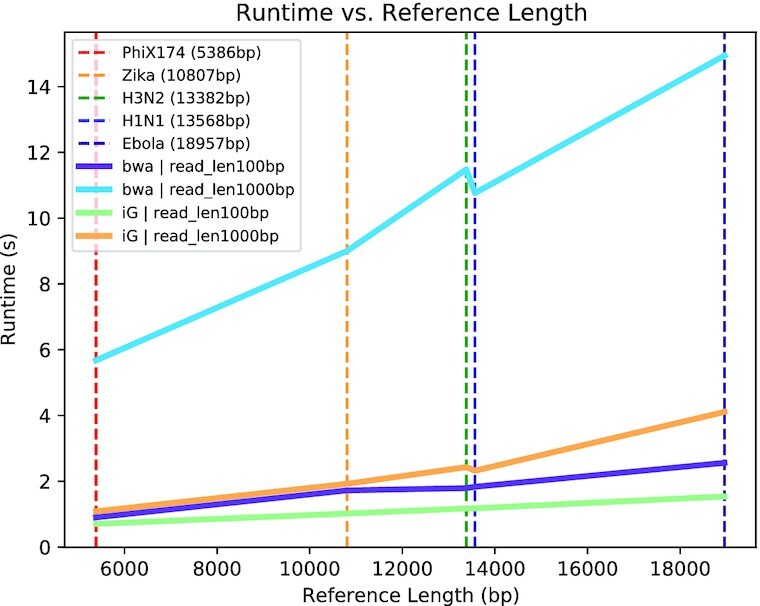
Runtimes for simulated reads from 5 reference genomes. The datasets consisted of reads averaging 100× coverage and a reference file. Each dataset was tested, defined as aligning then variant calling, using iGenomics running on an iPhone and a BWA/SAMtools pipeline running on a laptop. The technical specifications of the iPhone and laptop used for testing are described in the Results section. Each trend line indicates the runtime for each dataset using the denoted alignment and analysis software; iG indicates iGenomics and bwa indicates the BWA/SAMtools pipeline. The dotted lines indicate the specific measurements recorded.

When comparing the runtime of iGenomics against datasets with different genome lengths, we observe a nearly linear relationship between genome length and alignment runtime (Fig. 2). This is explained by a powerful feature of the BWT in which the time for an alignment of a single read is essentially independent of genome size. Consequently, because the simulations use a consistent amount of coverage per genome, the linear increase in runtime is explained by the linear increase in the number of reads to align. It is also worth noting that the iGenomics trend lines closely follow the pattern of those of BWA-MEM + SAMtools. This adds credibility both to iGenomics as a sequence alignment and analysis tool and to the field of portable genomics because all of these important viruses can be analyzed in <5 seconds on a mobile device.

To further explore the performance of iGenomics, we also compared the BWA+SAMtools pipeline described above with that of Minimap2 [20] + SAMtools, using the exact same steps in SAMtools after the SAM file was generated by the respective alignment tool. For the simulated H1N1 reads with read length 100 bp, sequence error rate of 0.01 (1%), and mutation rate of 0.1 (10%), we found that the indexing and alignment time was insignificant compared with the amount of time spent on variant calling: the alignment time for BWA was 0.899 s (22.42% of the total runtime), 0.440 s for Minimap2 (12.39% of the total runtime), and 3.11 s for identifying variants by converting the SAM file to BAM (0.24 s), sorting the BAM file (0.24 s), identifying candidate variants in BCF format (2.62 s), and computing the final variant calls (0.01 s). Thus, while Minimap2 is noticeably faster than BWA, the majority of time is spent on variant calling.

### Simulated read accuracy analysis

We next evaluated the accuracy of iGenomics using reads simulated from the H1N1 Influenza genome (same sample as above). In each trial, we simulated a mean of 100× coverage for all combinations of the following sets of parameters: sequence error rates of 0.01, 0.1, and 0.2, mutation rates of 0.001, 0.01, and 0.1, and read lengths of 100, 250, and 1,000 bp. Note that an error rate of 0.2 represents a 20% error rate and exceeds the current mean error rate for Nanopore sequencing [10]. The range of the simulation parameters is designed to test iGenomics across a variety of different possible sets of reads with which iGenomics could be used. After simulating the read sets, each simulated sample was independently aligned to an H1N1 reference genome using iGenomics. For each sample, we recorded the runtime and the reported list of mutations found. To check the validity of the mutations found by iGenomics, the reported mutations were compared to the DWGSIM-generated list of simulated mutations. We then compared the variants reported by iGenomics to DWGSIM, allowing for ≤5 bp differences to account for ambiguity that can occur, especially indels within locally repetitive sequencing. Key metrics that were evaluated relative to DWGSIM were precision, recall, and F-score (the harmonic mean of precision and recall).

**Figure 3:**
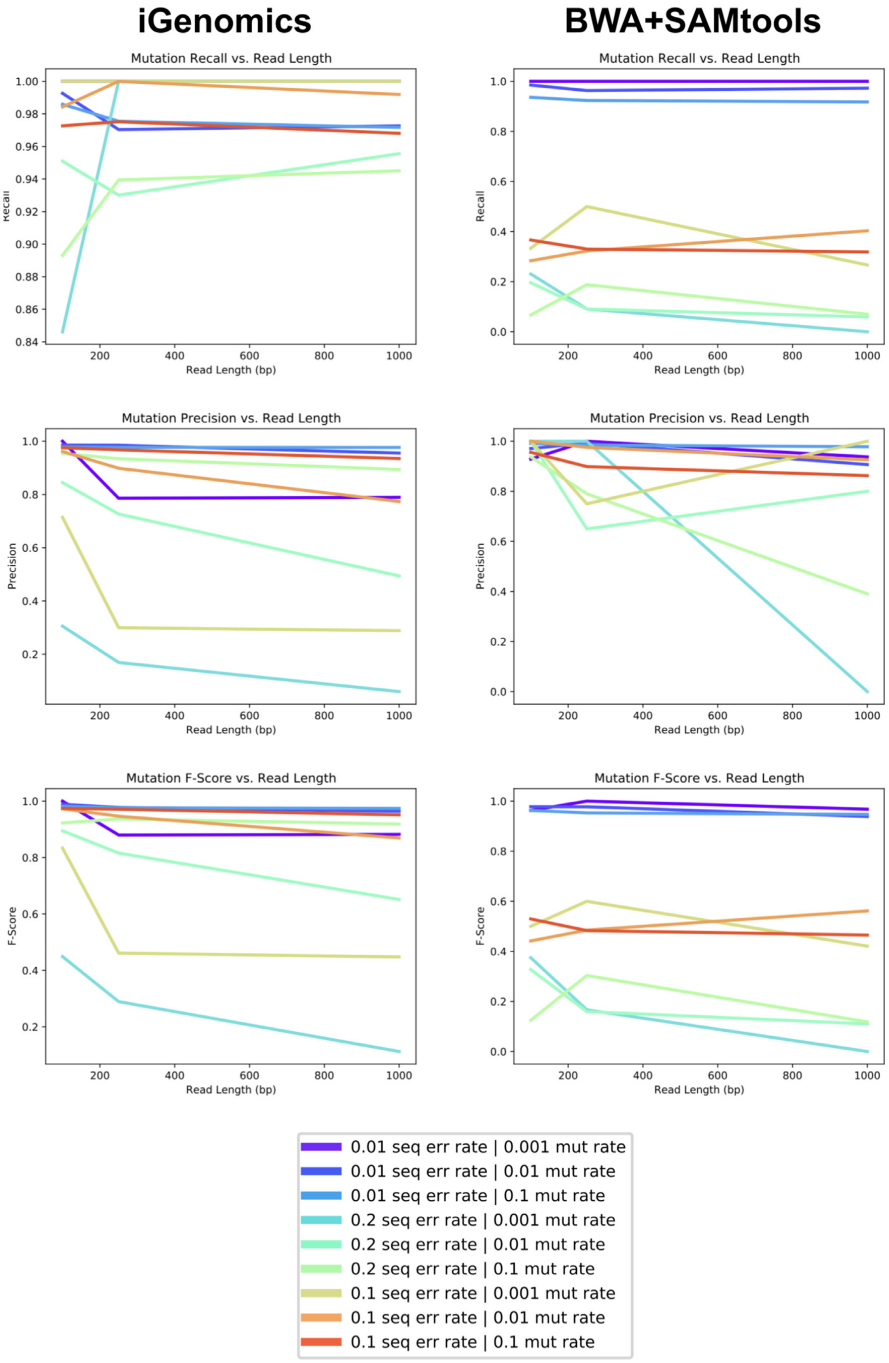
Mutation identification accuracy for simulated H1N1 flu datasets of varying mutation rates and error rates for iGenomics (left) and the BWA-MEM/SAMtools (right) pipeline. The top, middle, and bottom plots show recall, precision, and F-score, respectively.

The results of the comparisons between iGenomics’ reported mutations and DWGSIM's list of mutations confirm iGenomics' accuracy. Most datasets show a high degree of accuracy (F_1_) well over 90% (Fig. 3). The few experiments with lower precision or recall occur with the most difficult scenarios of the highest sequencing error rate and the lowest mutation rate. For comparison, the same results were also computed with input from a BWA-MEM/SAMtools pipeline. Interestingly, iGenomics tends to exhibit a higher degree of recall, precision, and overall accuracy (Supplementary Fig. S1).

**Figure 4:**
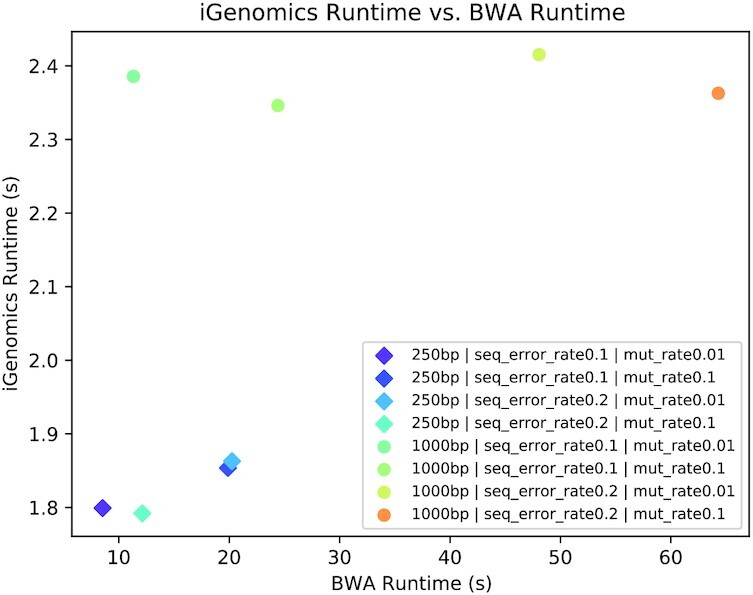
iGenomics runtime vs BWA/SAMtools pipeline runtime for simulated datasets of constant mutation rates and sequence error rates of H1N1 for varying read lengths.

Another important consideration for iGenomics is the runtime required. The runtime of iGenomics for each of these simulated datasets was <3 seconds (Fig. 2). Furthermore, iGenomics aligned reads and identified mutations in these simulated datasets ∼4–5 times faster than the BWA-MEM/SAMtools pipeline (Fig. 4). For context, the BWA-MEM/SAMtools runtime for these datasets was computed on an early 2015 MacBook Pro with a 2.9 GHz Intel Core i5 running OS X El Capitan while the iGenomics runtime was computed on a 2017 iPhone 8 with a 2.39 GHz A11 Bionic Chip running iOS 12.3.1. All timing results presented in this article use these hardware configurations, although we tested iGenomics on several iPhone and iPad models to ensure usability across screen sizes and system resources.

### Viral genome analysis

iGenomics was next tested on several clinical and environmental viral samples sequenced using the ONT MinION to demonstrate both the functionality and accuracy of iGenomics relative to standard tools such as BWA-MEM and SAMtools. The purpose of these tests is to show the overall utility of iGenomics as a mobile counterpart to desktop aligners and analysis software typically used by researchers and as a novel sequence analysis platform.

These tests focused on public MinION data from Ebola (sample [21] from [2]) and Zika (sample [22] from [23]), as well as MinION and MiSeq data from a clinical H3N2 sample that we previously collected (A/New York/A39/2015 (H3N2)) [24] (Methods). The Ebola trial focused on comparing mutations found by iGenomics to those found by SAMtools using the isolate Ebola virus/H.sapiens-wt/SLE/2014/Makona-G3686.1 as the reference (GenBank: KM034562.1). For Zika, the test was based on using a ground-truth set of mutations derived by comparing the consensus genome with nucmer [25] to the isolate Zika virus/H.sapiens-tc/KHM/2010/FSS13025 (GenBank: KU955593.1) as the reference. The H3N2 test was designed to demonstrate iGenomics' consistency across data produced by different sequencers by comparing the results of the Nanopore and MiSeq data when aligning to the isolate (A/California/7/2004(H3N2)) genome.

In all of the cases examined, iGenomics had a faster runtime than the desktop alignment pipeline of BWA-MEM/SAMtools (Table 1). This is likely due to a difference in how iGenomics and the desktop software store the alignments in memory. Because iGenomics is targeted to be a focused mobile analysis platform for small genomes, it needs to run very rapidly. Instead of separately reporting each alignment and writing the alignments to disk, then separately sorting the alignments, and then scanning for variations, as BWA-MEM/SAMtools does, iGenomics records the full gapped alignments and coverage profile matrix in RAM so that the subsequent mutation identification can avoid repeating computations. Furthermore, iGenomics keeps these data in RAM until the user exits the analysis screen to allow for exploring the various visualizations and performing interactive analysis with negligible lag time. This presents a standard time vs RAM trade-off present in many software applications, and here we have elected for fast processing to ensure that the application is as responsive as possible.

**Table 1:** Comparison between iGenomics and BWA-MEM/SAMtools pipeline for real reference genomes and reads obtained from MinION (Nanopore) and MiSeq sequencers

Parameter	iGenomics*	BWA + SAMtools	iGenomics*	BWA + SAMtools
	**MinION Ebola data**		**MinION Zika data**	
Alignment rate (%)	99.24	100	81.46	94.11
Runtime (seconds)	24.71	428.96	13.11	189.19
Precision, recall, accuracy (%)				
Compared with SAM calls^⁺^	61.54, 66.67, 64.00	N/A	86.79, 79.77, 83.13	N/A
Compared with nucmer calls^⁺^	N/A	N/A	86.16, 88.96, 87.54	83.24, 93.51, 88.07
	**MinION H3N2 data**		**MiSeq H3N2 data**	
Alignment rate (%)	99.36	98.08	98.18	98.58
Runtime (seconds)	28.04	180.49	4.78	27.59
Precision, recall, accuracy (%)				
Compared with SAM MinION calls	40.24, 93.44, 56.25	N/A	N/A	N/A
Compared with SAM MiSeq calls^⁺⁺^	74.82, 87.12, 80.50	99.45, 49.86, 66.42	99.73, 99.73, 99.73	N/A

* Unreported heterozygosity is present in the mutations called.

⁺ This method of variant calling is considered to be the ground-truth. BWA+SAMtools has an N/A (not applicable) in these cells because the BWA + SAMtools output is considered the ground-truth.

⁺⁺ This method of variant calling is considered to be the ground-truth.

### Influenza typing

Influenza disease is caused by RNA viruses from the family Orthomyxoviridae [26]. There are 3 distinct viral types, A, B, and C, that can infect humans. Influenza types A and B cause the annual epidemics, while influenza C is generally less severe. The influenza A genome is organized into 8 segments and is classified into subtypes based on genetic variants within the 2 proteins on the surface of the virus: hemagglutinin (H) and neuraminidase (N). There are 18 different hemagglutinin subtypes and 11 different neuraminidase subtypes (H1–H18 and N1–N11, respectively). Many of the major influenza pandemics have been caused by influenza type A infections. For example, the 1918 flu pandemic (the “Spanish flu”), was caused by a deadly Influenza A virus strain of subtype H1N1, and the Hong Kong Flu in 1968 was caused by the H3N2 subtype. Consequently, the type and subtype of an unknown influenza sample are extremely important and urgent to determine.

As a final demonstration of how iGenomics can be used, we also considered an influenza identification task where influenza sequencing data are aligned to several strains of flu at the same time in an attempt to determine the type and subtype. For this, we developed an influenza “pan-genome reference sequence” containing representatives for 3 different influenza genomes related to antigenic strains that were circulating from 2009 to 2016: (H1N1)pdm09 (A/California/04/2009), H3N2 (A/Brisbane/10/2007, A/Perth/16/2009, A/Texas/50/2012, A/Victoria/361/2011, and A/NewYork/03/2015), and Influenza B (B/New York/1352/2012). For this analysis, segments that are shared across Influenza A subtypes were only reported once. For the pan-genome, we also include a catalog of mutations in these genomes that have specific variants known to reduce the efficacy of antiviral treatments. The identity of the A segment is identified by evaluating which of the potential segment types has the largest number of alignments. In the context of iGenomics, the pan-genome approach is preferable to aligning the reads against multiple Influenza genomes in isolation because it is much simpler and allows for typing and variant identification at the same time. Worth noting, the pan-genome approach does not sacrifice accuracy or performance, as shown below.

To test alignments against the pan-genome, we ran iGenomics using simulated MinION (1,000 bp, sequence error rate 10.0%) and Illumina (100 bp, sequence error rate 1.0%) reads from pH1N1 and H3N2 with mutation rates 0, 0.001, and 0.005. After alignment, we evaluated whether the reads were correctly aligned to the type and subtype from which they originated. If the alignment matches the segment of origin, we consider that alignment “passing.” The segment identification rate is the number of passing alignments divided by the total number of alignments. The results of this experiment show that we have a >93% identification rate, meaning that in most cases this simple process can accurately and quickly determine the type and subtype of the flu genome entirely on a mobile device (Table 2).

**Table 2:** Alignment details for simulated datasets aligned using iGenomics to a pan-genome composed of multiple Influenza genomes

Parameter	MinION simulated data		Illumina simulated data	
	pH1N1	H3N2	pH1N1	H3N2
Alignment rate (%)	100	100	100	99.84
Runtime (seconds)	4.25	4.17	1.60	1.63
Segment identification rate (%)^+^	99.11	95.04	99.84	93.02

The pH1N1 reads were simulated from the (H1N1)pdm09 (A/California/04/2009) genome and the H3N2 reads were simulated from the H3N2 (A/NewYork/03/2015) genome.

⁺ Segment identification rate is the number of alignments that aligned to the correct reference within the pan-genome divided by the total number of alignments.

## Discussion

DNA sequencing has advanced tremendously over the past 3 decades; a process that once required hundreds of millions of dollars can now be done on handheld devices costing only $1,000 [27]. However, it is important to consider that sequenced DNA reads themselves provide little information without software to align and analyze them. For high-end servers and laptops, this software already exists; for mobile devices, iGenomics is the first comprehensive solution for researchers and citizen scientists to easily analyze sequence data.

iGenomics can be used in virtually any location because of the inherent portability of mobile devices like the iPad and iPhone. iGenomics implements the same advanced bioinformatics algorithms that are used for rapid alignment and analysis for other platforms. Consequently, the true novelty of this application is not in the algorithms used but rather how they have been implemented in a mobile environment. The entire workflow for iGenomics is designed to be simple and intuitive. A user effortlessly picks a reads file to analyze and, once selected, the alignment, variant calling, and visualization are completed within seconds. This is accomplished without any internet connectivity through an optimized implementation in Objective-C.

iGenomics is designed for quickly computing detailed genetic information about specific mutations within different viral or bacterial genomes. An important use case of iGenomics could be a researcher with limited computational resources sequencing complementary DNA (cDNA) of a coronavirus sample, loading and aligning the cDNA reads with iGenomics, and getting a first analysis of the coronavirus mutations within a few seconds. To support this capability, we have developed a tutorial with the MinION reads (SRX7615629) and consensus genome (MN938384.1) from patient HKU-SZ-002a, as well as the consensus genome from a bat SARS-like coronavirus isolate (MG772934.1) previously used for comparisons [28, 29]. Following the tutorial, these data can easily be downloaded on one's iOS device and imported directly into iGenomics to be analyzed. Another promising capability of iGenomics is its ability to load reference genomes and reads from outside sources, perform alignment and variant calling, and export the results all without any internet access. For example, by using Airdrop to both import and export data from iGenomics, a researcher can analyze DNA in remote locations without any internet connectivity. Because the MinION uses a USB connection that is not available on an iPhone or iPad, users will first need to collect the raw sequencing data on their laptop or server, as well as use these platforms to base call the signal data into nucleotide sequences. However, once sequencers are available that can read DNA directly into iOS devices, iGenomics will work out of the box to allow for importing of this sequenced data, eliminating the requirement for a laptop in the end-to-end analysis pipeline.

Future developments for iGenomics are far-reaching as DNA sequencing instruments continue to evolve to the point where they could be directly attached or integrated with mobile devices. In fact, ONT has announced that they hope to have a new sequencer, named the “SmidgION,” that connects directly to iOS devices available for researchers in the near future [30]. At that point, using mobile sequencing technology with iGenomics, DNA can truly be sequenced, aligned, and analyzed anywhere and absolute mobility of the genomics field will be achieved. As the processing power and memory contained within mobile devices improves, so will the overall performance of iGenomics in handling even larger and more complex samples.

## Methods

The implementation of iGenomics follows the state-of-the-art algorithms and data structures used in standard bioinformatics applications. However, the visualization of the read alignments and mutations is unique to iGenomics and was created with the intention of allowing the user to have powerful analysis capabilities while still maintaining a simple mobile-friendly interface.

### Indexing the genome with BWT

**Figure 5:**
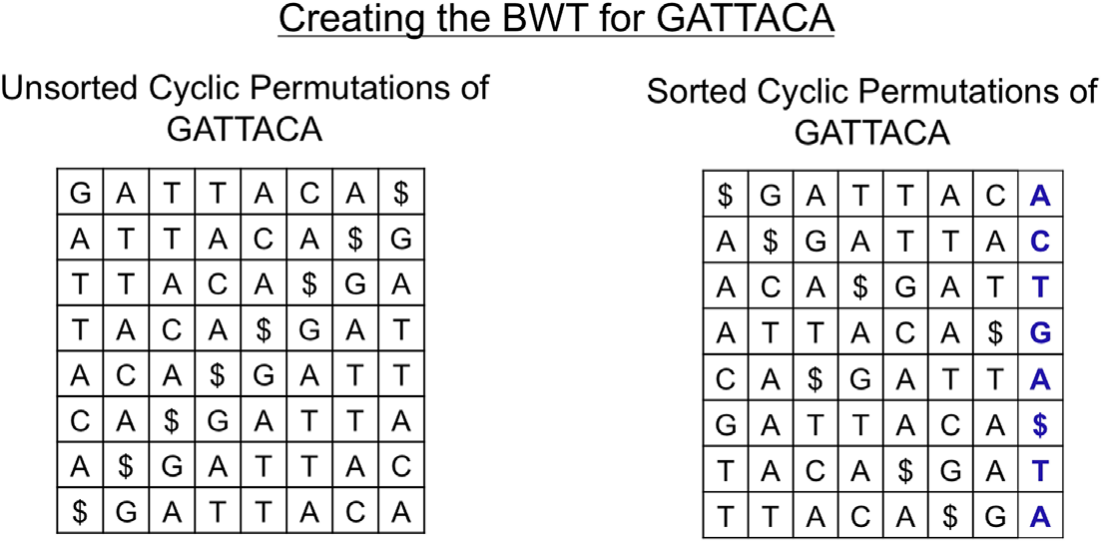
Diagram of how the Burrows-Wheeler transform is created. (Left) All cyclic permutations of the text “GATTACA.” (Right) The Burrows-Wheeler matrix of the text consisting of the sorted cyclic permutations of the text.

The BWT is constructed by lexicographically sorting the cyclic permutations of the input genome appended by an end-of-string character. By convention, we use a dollar sign as the end-of-string character, which has a lexicographical value less than any letter in the English alphabet and ensures that the end of the original sequence can be found. For example, the cyclic permutations of the string “CAT” with the end-of-string character “$” are “CAT$,” “AT$C,” “T$CA,” and “$CAT,” which can be sorted as “$CAT,” “AT$C,” CAT$,” and “T$CA.” This sorted list creates what is known as the Burrows-Wheeler matrix (BWM). Then, to compute the BWT from the sorted permutations, the last character of each row in the matrix is extracted in order and appended to a string (Fig. 5).

To first lexicographically sort the cyclic permutations, a quick and efficient sorting algorithm must be used so that this function is fully optimized. iGenomics uses a version of QuickSort, a divide-and-conquer sorting algorithm, because on average it takes O(*n* log *n*) time for *n* objects to be sorted. Although there are now some more efficient BWT construction algorithms [31], given that iGenomics is targeted towards relatively small genomes (<100,000 bp), the amount of time for BWT sorting is negligible compared to the time to align the reads. Finally, to obtain the BWT from the sorted array, the final character of each row in the matrix is copied into a string with the first character copied having the first position, the second character copied having the second position, and so forth.

### Read alignment

iGenomics uses a seed-and-extend process for read alignment in which first relatively short exact matches, known as seeds, are found using the BWT, after which they are then extended into end-to-end alignments using dynamic programming. The seed size is based on the maximum edit distance (a user-specified parameter) allowed for a read that successfully aligns to be considered a match. The maximum edit distance is inputted as a decimal value edit rate, and multiplying that value by the length of the given read will give the maximum possible edit distance we allow when aligning that read. During the aligning process, each read is split into the edit distance plus 1 segment of equal length. This relies on the widely used technique that if the string matches with ≤*X* edits, then ≥1/(*X* + 1) of the segments must still match without error [32]. For example, if the user allows only 1 edit, the algorithm divides the read into left and right halves [1/(1 + 1)] knowing that the correct alignment will include an exact match of 1 of those segments.

**Figure 6:**
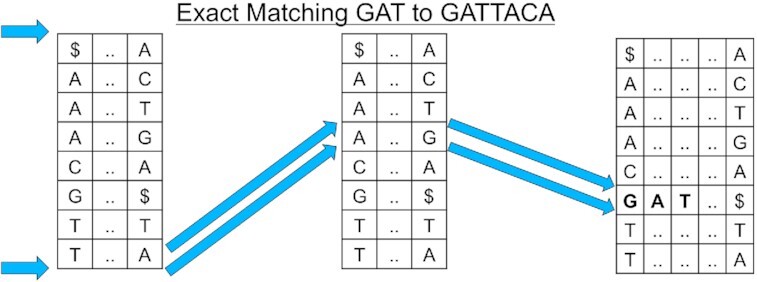
A diagram showing the exact match algorithm by repeated application of the last-first property using the characters of the query string.

Exact matching means finding all of the places in the reference genome where a given query matches exactly, character for character across its entire length (Langmead 2012) [33]. To do this effectively, the trait of the BWT known as the “last-first property" is used as the basis for an exact matching algorithm. The last-first property states that the occurrence of any character in the last column of the BWM, which is the BWT, corresponds to the same occurrence of that character in the first column of the BWM. Using the first column of the BWM and the BWT to create an FM-index, the algorithm navigates the rows of the index that contain exact matches and then converts these positions from the BWT to positions in the reference genome (Fig. 6).

**Figure 7:**
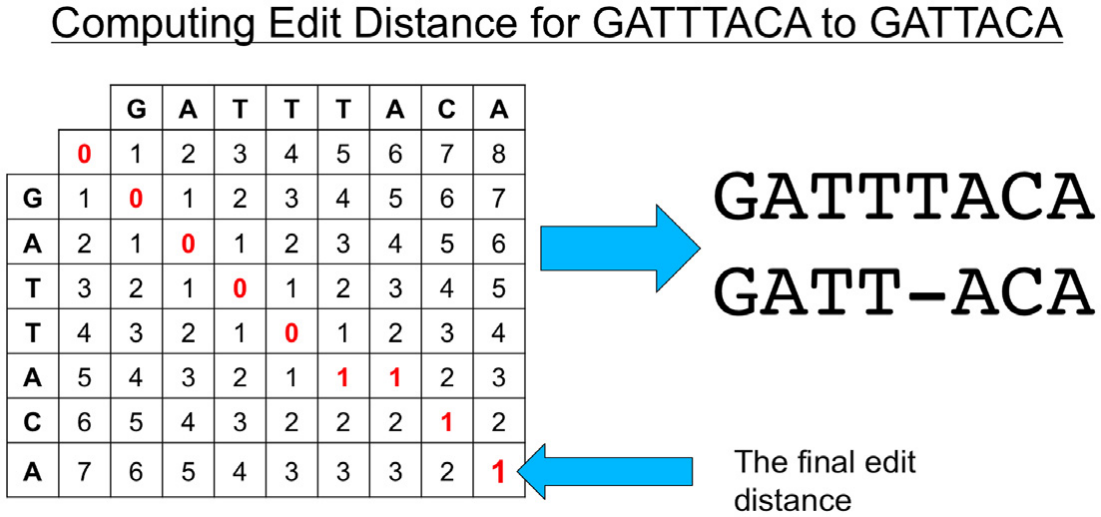
A diagram showing how edit distance is computed for 2 strings. Each cell of the matrix represents the minimum of 3 possible values: (1) the left cell plus 1 (representing the cost of adding a gap on the left string); (2) the upper cell plus 1 (representing the cost of adding a gap on the top string; and (3) the upper left cell plus 0, if the top string equals the left string, or 1, if the characters do not match to account for the cost of another substitution.

After the seeds are found, iGenomics computes the end-to-end edit distance allowing for substitutions as well as insertions and deletions [34] (Fig. 7). To make this as efficient as possible, iGenomics uses a banded computation. This method works by only computing a subset of the dynamic programming matrix, a band of the edit distance table, with the band having a standard width of (the maximum edit distance * 2 + 1). To determine where to begin the band computation, iGenomics attempts to exact-match a 20 bp substring of the read. A substring length of 20 bp was chosen because we found that it represented the optimal trade-off in terms of performance and reliability of identifying alignments. If the exact match is successful, the banded distance will be computed relative to the matched position of the substring. If the exact match is unsuccessful, an exact match with the 20 bp substring of the read starting at the second character will be attempted. This process continues with the substrings continuously moving 1 character over until either the read successfully aligns or none of the exact-matched 20 bp substrings yields a successful alignment.

### Coverage profile and variant identification

**Figure 8:**
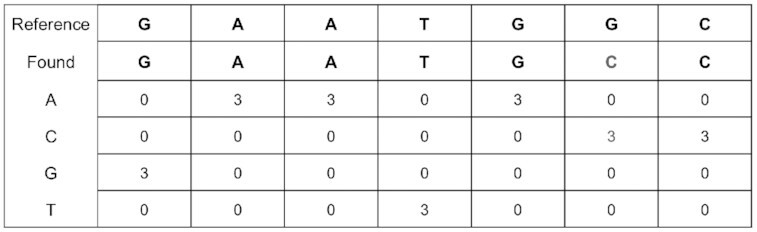
Table showing how the coverage profile is represented within iGenomics, summarizing how the reads align to the reference genome (an example of reads aligned to a reference genome is shown in Fig. 1). As can be seen in the sixth column, there is a mutation where the base C was found when the reference was base G.

The coverage profile concisely summarizes how the reads are aligned to the genome (Fig. 8). The internal data structure for the profile is a coverage profile matrix, which spans the genome and at each position contains a row for the number of matched base pairs, A, C, G, T, and (non–base pair) deletion characters. The matched positions of each read are tallied and the characters of the read are added, so that the positions of the matrix that the read overlaps are marked within the matrix. Once the coverage profile matrix is completely generated, variants can be identified, a graphical representation of the profile can be formed, and the number of alignments can easily be seen.

Variants are identified by scanning the array of matched characters, and at each position if the matched character differs from the reference character, a mutation, or variant, would be reported [15]. The major challenge of this analysis is distinguishing sequencing errors from real mutations, and differentiating between homozygous and heterozygous mutations. In a diploid genome, homozygous mutations are mutations that occur on both copies of a chromosome whereas heterozygous mutations occur on 1 copy of a chromosome but not both. iGenomics recognizes heterozygous mutations as positions in the genome where there is a nearly equal coverage of >1 base existing in the set of aligned reads according to a user-specified relative minimum heterozygosity threshold. Thus, if ≥2 bases at a position have relative coverages greater than that threshold, the mutation present at that position is considered to be heterozygous. In haploid species, such as the viral and bacterial pathogens described above, this threshold is used to find variants that occur within a minimum allele frequency within the population.

Immediately after alignment has completed, each position within the reference genome is assigned a value indicating whether the reads at that position matched either exactly, heterozygously, homozygously, heterozygously where there is a known mutation, or homozygously where there is a known mutation. This allows iGenomics to highlight all mutations with their associated heterozygosity and importance. Known mutations are loaded through a user-inputted text file. This file contains each known (important) mutation's reference base, mutated base, position, segment (or chromosome) that the mutation is expected to occur in, and a free-text description of what this mutation indicates. The known mutations functionality enables iGenomics to be specifically targeted for the analysis and treatment of different genomes, such as known mutations associated with influenza antiviral resistance.

### Visualizations and interactive analysis

The main challenge with the GUI was to create one that was both useful and unique when compared to other desktop DNA analysis software. The key to achieving these goals was to take advantage of the distinctive features of the iOS environment. Ultimately, a custom graphics engine was built to handle the constant redrawing of the analysis interface, and, visually, this engine sits on top of Apple's CoreGraphics library. In addition to the analysis interface, a utility interface was developed, which contains features for rapidly analyzing and quickly navigating the alignments.

The solution to developing this interactive analysis screen was to use many touch-related functions that are natural to anyone who has ever used a touch screen mobile device (Supplementary Figs S2–S8). Scrolling requires a simple finger drag, while viewing a large-scale version of the coverage profile merely requires performing a pinch gesture on the screen. The information pertaining to mutations can be viewed at any position by tapping on one of the reference genomes or found genome boxes at that position. Even this action takes advantage of the mobile iOS environment because a popover view is used to display the information at the tapped position. At the bottom of the screen, there is a variable scrubbing speed slider so that the user can move across the genome quickly or at a slower rate by dragging up while moving the slider.

Simple functions such as searching for a specific query or position are also included in the analysis view. To minimize clutter on the screen, when a user searches for a certain string, he/she is instantly taken to the next occurrence of that string, as opposed to displaying a large list of positions to the user. One of the most notable of these functions is the ability to change the minimum relative heterozygosity value (known as mutation coverage within iGenomics) on the fly through a slider. Once the user has concluded analyzing on the mobile device, he/she has the option to export mutations and analysis data via a variety of means: e-mail, Dropbox, Airdrop, or sharing via installed apps (such as Google Drive). The mutations are outputted in a VCF file format so that they are compatible with traditional desktop analysis software.

### Flu isolate sequencing

#### Sample collection and amplification

Clinical specimens of nasopharyngeal swabs were collected from patients in New York City during the 2014–2015 flu season as previously described [24]. The specimen used in this study was designated as A/New York/A39/2015 (H3N2) and is available in the SRA as sample ID SAMN08454624. Briefly, the RNA was eluted in 30 µL of RNase-free water and 3 µL was used as a template for the amplification of the entire influenza A or B genome using a previously described multi-segment RT-PCR method [35]. The presence of the cDNA copies of the genomic segments was examined by running 3 µL of the multi-segment RT-PCR amplicons on a 0.8% agarose electrophoresis gel. The influenza genomic amplicons were purified using a 1x Agencourt AMPure XP purification step (Beckman Coulter, Indianapolis, IN), and assessed by Qubit analysis (ThermoFisher, Waltham, MA) to quantify the mass of the double-stranded cDNA present.

#### Nanopore MinION sequencing

The library preparation and sequencing procedures were performed following manufacturer's instructions for the Nanopore Sequencing using the SQK-MAP006 kit (Oxford Nanopore Technologies, Oxford, UK). Purified DNA was used for end repair and dA-tailing, followed by 1× AMPure XP beads purification. The resultant DNA was quantitated by Qubit analysis and the molarity was further determined by using the Agilent 2200 TapeStation system with a Genomic DNA ScreenTape (Agilent, Santa Clara, CA). Next, 0.2 pmol of the DNA was used in adaptor ligation, and the reaction was purified using MyOne C1-beads. The final DNA was eluted in 25 µL Elution Buffer and is called Pre-sequencing Mix. For the SQK-MAP006 sequencing kit, 12 µL Pre-sequencing Mix was combined with 75 μL 2× Running Buffer, 59 μL nuclease-free water, and 4 μL Fuel Mix and then loaded into the FLO-MAP003 flow cell. A re-loading was also performed. The sequencing was run on the MIN-MAP001 MinION sequencing device, which was controlled by the MinKNOW software using the MAP_48Hr_Sequencing_Run.py script provided by ONT or using the MAP_140to5xVoltage_Tuned_plus_Yield_Sequencing_Run.py script provided by John Tyson of the University of British Columbia. Raw data were uploaded to the cloud-based Metrichor platform and base-calling was performed using the application of 2D Basecalling for SQK-MAP005 Rev 1.62 or 2D Basecalling for SQK-MAP006 Rev 1.62.

#### Illumina MiSeq sequencing

The sample was prepared for sequencing on the Illumina MiSeq platform (Illumina, San Diego, CA) according to the manufacturer's protocol (15,039,740 v01) as previously described [24]. Sequencing data were then generated by a 2 × 300 bp run using an Illumina MiSeq 600 Cycle v3 reagent kit.

## Availability of Supporting Source Code and Requirements

Project name: iGenomics

Project home page: https://github.com/stuckinaboot/iGenomics

Operating system(s): iOS

Programming language: Objective-C

License: MIT License

Other requirements: Precompiled binary is for free on Apple's App Store (https://apple.co/2HCplzr)

RRID:SCR_019142

## Data Availability

All sequencing data (genuine and simulated) along with a tutorial on iGenomics are available at http://schatz-lab.org/iGenomics/. We have also archived all of these data along with the code for reproducing the results from this article in the *GigaScience* Database, GigaDB [36].

## Additional Files

**Supplementary Figure S1**. Mutation identification accuracy for simulated H1N1 flu datasets. Plots show the accuracy of varying mutation rates and read length for iGenomics (left) and the BWA-MEM/SAMtools (right) pipeline. The results were computed in the same manner as described in the third section of the Results (Simulated read accuracy analysis): the simulated reads consisted of H1N1 read sets simulated with a mean coverage value of 100 and for all combinations of the following sets of parameters: sequence error rates of 0.01, 0.1, and 0.2, mutation rates of 0.001, 0.01, and 0.1, and read lengths of 100, 250, and 1,000 bp.

**Supplementary Figure S2**. iGenomics reference selection. (Left) launch screen, (middle) file selection page, (right) individual file selector. By pressing the “Start” button on the launch screen, the user is brought to the file selection page. Pressing “Select File” on the file selection page will allow the user to use the individual file selector to choose a default file (pre-packaged with iGenomics) or imported file (saved to iGenomics from an external app) or to use Dropbox's UI to choose a file from the user's Dropbox account. Additionally, the user can select “Analyze,” which will immediately begin to align the input reads to the reference using the most recently used parameters, or “Configure,” which will present the parameter selection page.

**Supplementary Figure S3**. iGenomics alignment parameter selection. (Left) parameter selection page with trimming disabled, (middle) parameter selection page with trimming enabled, (right) computing page. From the file selection page in Supplementary Fig. S2, if the user chooses “Analyze,” the right computing page will be shown and if the user chooses “Configure,” the parameter selection page will be shown with the last parameters used. Pressing “Start Aligning” from the parameter selection page will begin aligning the reads using the configured parameters. On the computing page, the percentage indicates the total percent of reads aligned and the time remaining indicates the estimated time remaining before the alignment and variant identification process completes.

**Supplementary Figure S4**. iGenomics summary views. (Top) view selection page, (middle) alignment details page, (bottom) segment selection page. The view selection page allows the user to view the alignments display and coverage profile (shown in Fig. 1) as well as the coverage histogram and found mutations list (shown in Supplementary Fig. S5). The alignment details page displays information about the alignments, including the reads and reference file names, percentage of reads that matched, and the number of mutations, and allows the user to search the reference genome and adjust the minimum relative heterozygosity value (known as mutation coverage within iGenomics). The segment selection page lets the user intuitively choose a particular segment in the reference genome for which to view alignment information. These 3 pages, in addition to a fourth page (the important mutations display shown in Fig. 1), can be navigated with just a swipe.

**Supplementary Figure S5**. iGenomics coverage histogram and mutation list. (Top) coverage histogram, (bottom) found mutations list. The coverage histogram displays a plot of the frequencies of each coverage value with a Poisson curve for context. In these screenshots, we used simulated H1N1 reads of length 100 bp with a mean coverage value of 100. The found mutations list displays the number of mutations identified in each segment and information about each of those mutations. By tapping the circle “i” icon, the user can navigate directly to the mutation in the coverage profile or alignments view (whichever was most recently used). Adjusting the mutation coverage slider in Supplementary Fig. S4 will affect the mutations that are displayed in this list.

**Supplementary Figure S6**. iGenomics read information. (Top) position information popover, (bottom) read alignment popover. The position information popover for a given position displays coverage details, heterozygosity, and, if present, insertion mutations. This popover can be invoked by double-tapping anywhere in the column for a position from within the alignments display or coverage profile. The read alignment popover shows specifically how a particular read aligned to the reference genome, and can be brought up from the alignments display by long-pressing an aligned read.

**Supplementary Figure S7**. iGenomics iPad alignment display. (Top left) alignments display, (top right) coverage profile, (bottom left) partially zoomed-out coverage profile, (bottom right) fully zoomed-out coverage profile. The iPad application for iGenomics strongly resembles that of the iPhone application for all views except the analysis ones. In the analysis view, alignment details are always visible at the top of pane and the alignments display/coverage profile is displayed below the details. As with the iPhone version of iGenomics, the user can switch between the alignments display and coverage profile and can zoom out of either to see the relative coverage at varying levels of granularity.

**Supplementary Figure S8**. iGenomics iPad analysis display. (Top) analysis utilities, (bottom) found mutations list. Tapping the 3-line icon (hamburger button) in the top left of the analysis view will bring up the analysis utilities, which contains the same capabilities as the iPhone version of iGenomics but presents views in iPad-native popovers rather than new full-screen pages. Tapping on any of these utilities, such as the “Mutation List,” will present the results in a popover.

## Abbreviations

bp: base pairs; BWM: Burrows-Wheeler matrix; BWT: Burrows-Wheeler transform; cDNA: complementary DNA; GATK: Genome Analysis Toolkit; GUI: graphical user interface; IGV: Integrative Genomics Viewer; ONT: Oxford Nanopore Technologies; RAM: random access memory; SARS: severe acute respiratory syndrome; SRA: Sequence Read Archive; UI: user interface; USB: Universal Serial Bus; VCF: Variant Call Format.

## Competing Interests

M.C.S. has received travel funding from Oxford Nanopore Technologies Limited.

## Funding

The project was supported in part by the US National Science Foundation award (DBI-1350041) to M.C.S..

**Author Contributions**

A.P. developed the software, performed the sequence analysis, and wrote the manuscript. B.Z. performed the sequencing, constributed to the analysis, and wrote the manuscript. E.G. oversaw the sequencing, contributed to the analysis, and wrote the manuscript. M.C.S. oversaw the project, contributed to the analysis, and wrote the manuscript. All authors read and approved the final manuscript.

## Supplementary Material

giaa138_GIGA-D-20-00070_Original_Submission

giaa138_GIGA-D-20-00070_Revision_1

giaa138_GIGA-D-20-00070_Revision_2

giaa138_Response_to_Reviewer_Comments_Original_Submission

giaa138_Response_to_Reviewer_Comments_Revision_1

giaa138_Reviewer_1_Report_Original_Submission Stefan Prost -- 5/3/2020 Reviewed

giaa138_Reviewer_1_Report_Revision_1 Stefan Prost -- 10/11/2020 Reviewed

giaa138_Reviewer_2_Report_Original_Submission Yulia Suvorova -- 5/12/2020 Reviewed

giaa138_Reviewer_3_Report_Original_Submission Marcos Colebrook -- 5/13/2020 Reviewed

giaa138_Reviewer_3_Report_Revision_1 Marcos Colebrook -- 5/13/2020 Reviewed

giaa138_Supplemental_File
