# Supplementary material for: iGenomics: Comprehensive DNA sequence analysis on your Smartphone
Source: Gigascience. 2020 Dec 7;9(12):giaa138. doi: 10.1093/gigascience/giaa138 (PMC7720420; doi:10.1093/gigascience/giaa138)
Supplement: giaa138_GIGA-D-20-00070_Revision_1 [file giaa138_giga-d-20-00070_revision_1.pdf]

GigaScience
iGenomics: Comprehensive DNA Sequence Analysis on your Smartphone
--Manuscript Draft--

**Manuscript Number:**	GIGA-D-20-00070R1
**Full Title:**	iGenomics: Comprehensive DNA Sequence Analysis on your Smartphone
**Article Type:**	Research
**Funding Information:**	Directorate for Biological Sciences (DBI-1350041) — Dr. Michael Schatz
**Abstract:**	Following the miniaturization of integrated circuitry and other computer hardware over the past several decades, DNA sequencing is following a similar path. Leading this trend is the Oxford Nanopore sequencing platform, which currently offers the hand-held MinION instrument and even smaller instruments on the horizon. This technology has been used in several important applications, including the analysis of genomes of major pathogens in remote stations around the world. However, despite the simplicity of the sequencer, an equally simple and portable analysis platform is not yet available.<br><br>iGenomics is the first comprehensive mobile genome analysis application, with capabilities to align reads, call variants, and visualize the results entirely on an iOS device. Implemented in Objective-C using the FM-index, banded dynamic programming, and other high-performance bioinformatics techniques, iGenomics is optimized to run in a mobile environment. We benchmark iGenomics using a variety of real and simulated Nanopore sequencing datasets of viral and bacterial genomes and show that iGenomics has performance comparable to the popular BWA-MEM/Samtools/IGV suite, without needing a laptop or server cluster. iGenomics is available open-source (https://github.com/stuckinaboot/iGenomics) and for free on Apple's App Store (https://apple.co/2HCplzr).
**Corresponding Author:**	Michael Schatz<br>Johns Hopkins University<br>Baltimore, MD UNITED STATES
**Corresponding Author Secondary Information:**	
**Corresponding Author's Institution:**	Johns Hopkins University
**Corresponding Author's Secondary Institution:**	
**First Author:**	Aspyn Palatnick
**First Author Secondary Information:**	
**Order of Authors:**	Aspyn Palatnick<br>Bin Zhou<br>Elodie Ghedin<br>Michael Schatz
**Order of Authors Secondary Information:**	
**Response to Reviewers:**	Please see the response letter PDF file.
**Additional Information:**	
**Question**	**Response**
Are you submitting this manuscript to a special series or article collection?	No
**Experimental design and statistics**	Yes

Full details of the experimental design and statistical methods used should be given in the Methods section, as detailed in our Minimum Standards Reporting Checklist. Information essential to interpreting the data presented should be made available in the figure legends.<br><br>Have you included all the information requested in your manuscript?	
**Resources**<br><br>A description of all resources used, including antibodies, cell lines, animals and software tools, with enough information to allow them to be uniquely identified, should be included in the Methods section. Authors are strongly encouraged to cite Research Resource Identifiers (RRIDs) for antibodies, model organisms and tools, where possible.<br><br>Have you included the information requested as detailed in our Minimum Standards Reporting Checklist?	Yes
**Availability of data and materials**<br><br>All datasets and code on which the conclusions of the paper rely must be either included in your submission or deposited in publicly available repositories (where available and ethically appropriate), referencing such data using a unique identifier in the references and in the "Availability of Data and Materials" section of your manuscript.<br><br>Have you have met the above requirement as detailed in our Minimum Standards Reporting Checklist?	Yes

Powered by Editorial Manager® and ProduXion Manager® from Aries Systems Corporation

Manuscript Click here to access/download;Manuscript;Manuscript.pdf

iGenomics: Comprehensive DNA Sequence Analysis on your Smartphone

Aspyn Palatnick[1,2,3], Bin Zhou[4], Elodie Ghedin[4,5,*], Michael C. Schatz[2,7,†]

[1]Cold Spring Harbor High School, Cold Spring Harbor, NY 11724
[2]Simons Center for Quantitative Biology, Cold Spring Harbor Laboratory, Cold Spring Harbor, NY 11724
[3]University of Pennsylvania, Philadelphia, PA 19104
[4]Department of Biology, New York University, New York, NY 10003
[5]Department of Epidemiology, School of Global Public Health, New York, NY 10003
[6]Departments of Computer Science and Biology, Johns Hopkins University, Baltimore MD, 21211

[*] Current Affiliation: National Institute of Allergy and Infectious Disease, National Institutes of Health, Bethesda, MD, 20892
† contact: mschatz@cs.jhu.edu

Abstract

Following the miniaturization of integrated circuitry and other computer hardware over the past several decades, DNA sequencing is following a similar path. Leading this trend is the Oxford Nanopore sequencing platform, which currently offers the hand-held MinION instrument and even smaller instruments on the horizon. This technology has been used in several important applications, including the analysis of genomes of major pathogens in remote stations around the world. However, despite the simplicity of the sequencer, an equally simple and portable analysis platform is not yet available.

iGenomics is the first comprehensive mobile genome analysis application, with capabilities to align reads, call variants, and visualize the results entirely on an iOS device. Implemented in Objective-C using the FM-index, banded dynamic programming, and other high-performance bioinformatics techniques, iGenomics is optimized to run in a mobile environment. We benchmark iGenomics using a variety of real and simulated Nanopore sequencing datasets of viral and bacterial genomes and show that iGenomics has performance comparable to the popular BWA-MEM/Samtools/IGV suite, without needing a laptop or server cluster. iGenomics is available open-source (https://github.com/stuckinaboot/iGenomics) and for free on Apple's App Store (https://apps.apple.com/us/app/igenomics-mobile-dna-analysis/id1495719841).

Background

DNA sequencing technology has made tremendous progress over the past 30 years (Goodwin, McPherson, and McCombie 2016). The earliest automated approaches, beginning with the capillary-based Sanger sequencing devices in the 1980s, were large bench-top instruments requiring extensive sequencing facilities to prepare and sequence the DNA. In the 2000s, high throughput second-generation sequencing instruments advanced the field with more compact and simpler designs. However, these advances have been limited in their reach, because they are not readily accessible by most individual laboratories and citizen scientists. Most substantially, the most widely used alignment and analysis tools are not targeting citizen scientists and require expert knowledge on using the command line to install several software packages, run the tools, and understand a variety of file formats.

Within the past few years, Oxford Nanopore Technologies (ONT, Oxford, UK) has introduced a small inexpensive hand-held sequencing instrument that has made it possible to perform genomics experiments with minimal facilities and in essentially any environment. Because of its small size,

Nanopore sequencing has been used in several environments that would be unthinkable for alternative instruments as diverse as monitoring the Ebola outbreaks in remote areas of Africa (Quick et al. 2016), monitoring Zika outbreaks in South America (Giovanetti et al. 2020), exploring reptile specimens in the rainforest (Pomerantz et al. 2018), and even on the International Space Station (Castro-Wallace et al. 2017). Nanopore sequencing has also played an important role in monitoring the transmission of SARS-COVID-19 around the world (Viehweger et al. 2019; Oude Munnink et al. 2020; Thielen et al. 2020). Nanopore sequencing technology works by measuring the change in ionic current as a DNA molecule is passed through a nanopore (Goodwin, McPherson, and McCombie 2016). The DNA molecules are typically a few hundred to tens of thousands of nucleotides long and the longest reported read has exceeded 2 million nucleotides (Payne et al. 2019). Once sequenced, the raw signal data are base-called into nucleotide strings called reads (Wick, Judd, and Holt 2019), which are typically stored in fastq format and saved for further processing, especially read alignment and variant analysis.

Several algorithms are available for this analysis. Modern aligners, such as Bowtie (Langmead et al. 2009) or BWA-MEM (Li 2013), often use the Burrows-Wheeler Transform (BWT) (Burrows and Wheeler 1994) and the closely related FM-index (Ferragina and Manzini 2000) as their core indexing data structure. These new approaches are suited to large data sets because of their compact space requirements and fast alignment times. After alignment, variant calling platforms, such as Samtools (Li et al. 2009) or GATK (McKenna et al. 2010), systematically scan the alignments to find well supported variants in the sample using a statistical model to distinguish homozygous from heterozygous variants and rule out spurious sequencing errors. After this automated variant identification, priority variants are also often manually inspected using IGV (Robinson et al. 2011) and other genome browsers to review the evidence for the variant calls and further rule out false positives.

The standard approach for analyzing reads is to align the reads to a reference genome on high-end laptops, servers, or even supercomputers. While this is possible for those with access to these technologies, these requirements may be out of reach for many researchers and citizen scientists. Instead, iGenomics just requires the sequenced reads, which can be loaded from the phone itself, the internet, or else where, and can allow anyone to perform sequence analysis and mutation identification. As with other mobile applications (web browsing, email, social media, etc), iGenomics can be used in a variety of settings that would be awkward to perform with a larger laptop, and many users will also prefer the more intuitive user interface. Furthermore, there are many important scenarios where analyzing these data without high-end computing hardware is desirable, especially in remote environments. Interestingly, current iOS devices, including both iPads and iPhones, have significant computing resources, with clock speeds and onboard RAM approaching that of high-end laptop computers. That said, no standalone genomics analysis software is currently available for iOS devices.

Addressing this critical gap, we have developed iGenomics, an iOS application that allows anyone to easily align and analyze DNA sequences in a mobile environment. iGenomics utilizes the same high performance algorithms for read alignment and variant calling as mainstream software, although iGenomics marks the first time these algorithms have been implemented in a mobile iOS environment. Additionally, using the advanced user interface features available in iOS, iGenomics allows for interactive visualization and inspection of the read alignments and variant calls, and contains additional features for reviewing critical mutations of interest. For example, iGenomics comes bundled with a listing of critical mutations in the influenza A virus that indicate which antivirals are most likely to be ineffective (Hussain et al. 2017).

Due to the lower amount of processing power in mobile devices compared to high-end desktop computers or servers, iGenomics is limited in the size of the genome that can be processed. However, the implementations in iGenomics have been rigorously tested through direct comparisons with the BWA-MEM/Samtools framework for alignment and variant calling for viral and microbial genomes.

All alignment and analysis algorithms employed by iGenomics have been tested on both real and simulated datasets to ensure consistent speed, accuracy, and reliability of both alignments and variant calls. Consequently, iGenomics is leading the shift of DNA analysis software and sequencing tools towards mobile devices and marks a great leap forward towards widespread DNA analysis by non-bioinformatician students, researchers and citizen scientists. Furthermore, iGenomics is available open-source to facilitate mobile genomics technology research and, in turn, accelerate the speed at which this technology is developed.

Results

Interactive Sequence Analysis on your Smartphone

iGenomics brings a high level of interaction to DNA sequence analysis (**Figure 1**). Common touchscreen gestures allow for users to browse the alignment data in an easy-to-use and intuitive manner. This allows for the app to be used with almost no learning curve.

The first step of analysis is selecting the reads and a reference genome for analysis in either fasta or fastq format. iGenomics provides multiple options for inputting both reads and reference files: selecting from a variety of default files for common bacterial genomes, using Dropbox to choose a file, or loading a fasta or fastq file straight into iGenomics from another app such as Google Drive, Files, or Airdrop. Then, from a single view, the user can choose the reads file, the reference file, and, optionally, a tab-delimited file annotating known important mutations. For example, iGenomics comes with a preloaded known mutations file that indicates certain mutations in the influenza genome, which, if present, cause resistance to certain antivirals (Hussain et al. 2017). This single view design is meant to be simplistic and requires minimal user effort. After choosing the files to align, the user can either select the "Analyze" button to align reads to the reference genome using the default parameters or can choose to configure certain parameters before aligning. The parameters available include the maximum error rate for alignments and to enable trimming for fastq files.

After aligning completes, the user is brought to the analysis pane. The main view, known as the alignments display, is an IGV-like rendering of how the reads are aligned to a reference genome, with the ability to scroll left, right, up, and down through all of the aligned reads. Aligned bases that differ from the reference base are highlighted in a different color, as are consensus calls. A long-touch on a read presents additional details about the read, including the read name, the edit distance of the alignment, the gapped read and gapped substring of the reference genome the read aligned to, and whether the forward read or the reverse complement aligned. The user can also use the pinch-gesture to zoom out, revealing a high-level overview of the individual alignments as well as a coverage profile of the number of reads that aligned at each position. Mutations are still highlighted after zooming out, allowing the user to see where all of the mutations occur in one view.

Another view within the analysis pane is the coverage profile, which displays the count of each base that aligned at each position. Positions where the reference base does not match the base of the reads are highlighted so that the user can see that this position contains a mutation (heterozygous mutation are highlighted with a different color). To scroll through the coverage profile, the user simply has to swipe left or right. If a user would like to view more detailed information about a given position, he/she simply holds down any of the boxes in that position and an informative view elaborating upon the position's contents will pop up. By using the pinch gesture to zoom-out, the user reveals a graph of the number of reads that aligned at each position, resembling that of the zoomed-out alignments display but with a full-screen graph.

The Summary window, accessible from within the analysis pane, has four pages and provides some useful tools for a high-level overview of the data. The first page provides buttons to view the alignments display, coverage profile, coverage histogram, and list of all found mutations. The coverage histogram graphs the frequency of each level of coverage, specifically the frequency of a

particular number of reads aligned to a position, and is overlaid by a Poisson curve for context. Within the list of all found mutations, the user can scroll through all mutations, and then select one to inspect that position in the analysis pane. The second page gives an overview of the alignments, including the percent of reads matched, the total number of reads input, the number of mutations, and the names of the reads and reference files. This page also provides the user with the capability to search for positions in the reference genome by position or by a query string, which uses BWT exact match for rapid searching. The third page contains a large picker view that allows the user to intuitively move between sequences/segments in the reference genome. The last page contains a list of known mutations if the user selected a known mutations file during the file input stage. This list contains mutation position, mutation details (such as resistance to antivirals), and a color-coded indicator denoting if a mutation was found at that position and if that mutation indicates a known mutation.

Simulated read runtime analysis

In order to observe the efficiency and accuracy of iGenomics running on an iPhone 8, we first tested several simulated data sets. The reference genomes we used were:

(1) phiX174, a widely used control sequence for Illumina sequencing (Genbank:NC_001422.1, 5386 bp);

(2) a Zika virus genome (isolate Zika virus/H.sapiens-tc/KHM/2010/FSS13025, 10807 bp);

(3) a H3N2 influenza genome (A/California/7/2004(H3N2), 13382 bp);

(4) a H1N1 influenza genome (A/New York/205/2001(H1N1), 13568 bp); and

(5) an Ebola genome (isolate Ebola virus/H.sapiens-wt/SLE/2014/Makona-G3686.1, 18957 bp).

From these reference genomes, we then simulated reads using DWGSIM (https://github.com/nh13/DWGSIM) according to the following conditions: the average coverage is 100x, the genetic mutation rate was set to 0.5% and the read characteristics would mirror reads produced by real-world sequencers. Accordingly, reads of length 100bp and sequence error rate of 1.0% were simulated to mirror reads generated by Illumina sequencers and reads of length 1,000bp and sequence error rate of 10.0% were simulated to mirror reads generated by Oxford Nanopore sequencers. Sequencing errors were introduced at random to mimic the errors produced by sequencers. For comparison purposes, we also measured the runtime when aligning and identifying variations using a BWA-MEM (Li 2013) using “-x ont2d” and Samtools pipeline for the same datasets. Notably, iGenomics uses an FM-index and banded dynamic programming implementation similar to BWA-MEM allowing the analysis to focus on major differences in hardware.

When comparing the runtime of iGenomics against datasets with different genome lengths, we observe a nearly linear relationship between genome length and alignment runtime (**Figure 2**). This is explained by a powerful feature of the BWT in which the time for an alignment of a single read is essentially independent of genome size. Consequently, since the simulations use a consistent amount of coverage per genome, the linear increase in runtime is explained by the linear increase in the number of reads to align. It is also worth noting that the iGenomics trend-lines closely follow the pattern of those of BWA-MEM+Samtools. This both adds credibility to iGenomics as a sequence alignment and analysis tool and to the field of portable genomics, as all of these important viruses can be analyzed in under 5 seconds on a mobile device.

To further explore the performance of iGenomics, we also compared the BWA+SAMtools pipeline described above with that of Minimap2 (Li 2018) + SAMtools, using exact same steps in SAMtools after the SAM file was generated by the respective alignment tool. For the simulated H1N1 reads with read length 100bp, sequence error rate of 0.01 (1%) and mutation rate of 0.1 (10%), we found that the

indexing and alignment time was insignificant compared to the amount of time spent on variant calling: the alignment time for BWA was 0.899s (22.42% of the total runtime), 0.440s for Minimap2 (12.39% of the total runtime), and 3.11s for identifying variants by converting the SAM file to BAM (0.24 s), sorting the BAM file (0.24 s), identifying candidate variants in BCF format (2.62 s), and computing the final variant calls (0.01 s). Thus, while Minimap2 is noticeably faster than BWA, the majority of time is spent on variant calling.

Simulated read accuracy analysis

We next evaluated the accuracy of iGenomics using reads simulated from the H1N1 Influenza genome (same sample as above). In each trial, we simulated an average of 100x coverage for all combinations of the following sets of parameters: sequence error rates of 0.01, 0.1, and 0.2, mutation rates of 0.001, 0.01, and 0.1, and read lengths of 100bp, 250bp, and 1,000bp. Note that an error rate of 0.2 represents a 20% error rate, and exceeds the current average error rate for Nanopore sequencing (Wick, Judd, and Holt 2019). The range of the simulation parameters is designed to test iGenomics across a variety of different possible sets of reads that iGenomics could be used with. After simulating the read sets, each simulated sample was independently aligned to an H1N1 reference genome using iGenomics. For each sample, we recorded the runtime and the reported list of mutations found. In order to check the validity of the mutations found by iGenomics, the reported mutations were compared to the DWGSIM-generated list of simulated mutations. We then compare the variants reported by iGenomics to DWGSIM, allowing for up to 5bp differences to account for ambiguity that can occur, especially indels within locally repetitive sequencing. Key metrics that were evaluated relative to DWGSIM were precision, recall, and F-Score (the harmonic mean of precision and recall).

The results of the comparisons between iGenomics' reported mutations and DWGSIM's list of mutations confirm iGenomics accuracy. Most datasets show a high-degree of accuracy (F1) well over 90% (**Figure 3**). The few experiments with lower precision or recall occur with the most difficult scenarios of the highest sequencing error rate and the lowest mutation rate. For comparison, the same results were also computed with input from a BWA-MEM/Samtools pipeline. Interestingly, iGenomics tends to exhibit a higher degree of recall, precision, and overall accuracy (**Supplemental Figure 1**).

Another important consideration for iGenomics is the runtime required. The runtime of iGenomics for each of these simulated data-sets was below 3 seconds (**Figure 2**). Furthermore, iGenomics aligned reads and identified mutations in these simulated datasets about 4x to 5x faster than the BWA-MEM/Samtools pipeline (**Figure 4**). For context, the BWA-MEM/Samtools runtime for these data sets was computed on an early 2015 MacBook Pro with a 2.9GHz Intel Core i5 running OS X El Capitan while the iGenomics runtime was computed on a 2017 iPhone 8 with a 2.39 GHz A11 Bionic Chip running iOS 12.3.1. All timing results presented in this paper use these hardware configurations, although we tested iGenomics on several iPhone and iPad models to ensure usability across screen sizes and system resources.

Viral Genome Analysis

iGenomics was next tested on several clinical and environmental viral samples sequenced using the Oxford Nanopore MinION in order to demonstrate both the functionality and accuracy of iGenomics relative to standard tools such as BWA-MEM and Samtools. The purpose of these tests is to show the overall utility of iGenomics as a mobile counterpart to desktop aligners and analysis software typically used by researchers and as a novel sequence analysis platform.

These tests focused on public MinION data from Ebola (sample https://raw.githubusercontent.com/nickloman/ebov/master/data/fastq/004674.2D.fastq from

(Quick et al. 2016)), and Zika (sample http://s3.climb.ac.uk/nanopore/primal_KX369547_R9.tgz from (Faria et al. 2016)), as well as MinION and MiSeq data from a clinical H3N2 sample we previously collected (A/New York/A39/2015 (H3N2)) (Ding et al. 2019) (**Methods**). The Ebola trial focused on comparing iGenomics found mutations to those found by Samtools using the isolate Ebola virus/H.sapiens-wt/SLE/2014/Makona-G3686.1 as the reference (GenBank: KM034562.1). For Zika, the test was based on using a ground-truth set of mutations derived by comparing the consensus genome with nucmer (Kurtz et al. 2004) to the isolate Zika virus/H.sapiens-tc/KHM/2010/FSS13025 (GenBank: KU955593.1) as the reference. The H3N2 test was designed to demonstrate iGenomics consistency across data produced by different sequencers by comparing the results of the Nanopore and MiSeq data when aligning to the isolate (A/California/7/2004(H3N2)) genome.

In all of the cases examined, iGenomics had a faster runtime than the desktop alignment pipeline of BWA-MEM/Samtools (**Table 1**). This is likely due to a difference in how iGenomics and the desktop software store the alignments in memory. Since iGenomics is targeted to be a focused mobile analysis platform for small genomes, iGenomics needs to run very rapidly. Instead of separately reporting each alignment and writing the alignments to disk, then separately sorting the alignments, and then scanning for variations, as BWA-MEM/Samtools does, iGenomics records the full gapped alignments and coverage profile matrix in RAM so that the subsequent mutation identification can avoid repeating computations. Furthermore, iGenomics keeps this data in RAM until the user exits the analysis screen to allow for exploring the various visualizations and performing interactive analysis with negligible lag time. This presents a standard time vs RAM tradeoff present in many software applications, and here we have elected for fast processing to ensure the application is as responsive as possible.

Influenza typing

Influenza disease is caused by RNA viruses from the family Orthomyxoviridae (Krammer et al. 2018). There are three distinct viral types, A, B, and C that can infect humans. Influenza types A and B cause the annual epidemics, while influenza C is generally less severe. The influenza A genome is organized into eight segments, and is classified into subtypes based on genetic variants within the two proteins on the surface of the virus: hemagglutinin (HA) and neuraminidase (NA). There are 18 different hemagglutinin subtypes and 11 different neuraminidase subtypes (H1 through H18 and N1 through N11, respectively). Many of the major influenza pandemics have been caused by influenza type A infections. For example, the 1918 flu pandemic (the "Spanish flu"), was caused by a deadly Influenza A virus strain of subtype H1N1, and the Hong Kong Flu in 1968 was caused by the H3N2 subtype. Consequently, the type and subtype of an unknown influenza sample is extremely important and urgent to determine.

As a final demonstration of how iGenomics can be used, we also considered an influenza identification task where influenza sequencing data are aligned to several strains of flu at the same time in an attempt to determine the type and subtype. For this, we developed an influenza "pan-genome reference sequence" containing representatives for three different Influenza genomes related to antigenic strains that were circulating from 2009 to 2016: H1N1pdm09 (A/California/04/2009), H3N2 (A/Brisbane/10/2007; A/Perth/16/2009; A/Texas/50/2012; A/Victoria/361/2011; and A/NewYork/03/2015), and Influenza B (B/New York/1352/2012). For this analysis, segments that are shared across influenza A subtypes were only reported once. For the pan-genome, we also include a catalog of mutations in these genomes that have specific variants known to reduce the efficacy of antiviral treatments. The identity of the A segment is identified by evaluating which of the potential segment types has the largest number of alignments. In the context of iGenomics, the pan-genome approach is preferable to aligning the reads against multiple Influenza genomes in isolation because it is much simpler and allows for typing and variant identification at the

same time. Worth noting, the pan-genome approach does not sacrifice accuracy or performance, as shown below.

In order to test alignments against the pan-genome, we ran iGenomics using simulated MinION (1,000bp, sequence error rate 10.0%) and Illumina (100bp, sequence error rate 1.0%) reads from pH1N1 and H3N2 with mutations rates 0, 0.001, and 0.005. After alignment, we evaluated if the reads were correctly aligned to the type and subtype that they originated from. If the alignment matches the segment of origin, we consider that alignment "passing". The segment identification rate is the number of passing alignments divided by the total number of alignments. The results of this experiment show that we have a greater than 93% identification rate, meaning that in most cases this simple process can accurately and quickly determine the type and subtype of the flu genome entirely on a mobile device (**Table 2**).

Discussion

DNA sequencing has advanced tremendously over the past three decades; a process that once required hundreds of millions of dollars can now be done on handheld devices costing only $1,000 (Shendure et al. 2017). However, it is important to consider that sequenced DNA reads themselves provide little information without software to align and analyze them. For high-end servers and laptops, this software already exists; for mobile devices, iGenomics is the first comprehensive solution for researchers and citizen scientists to easily analyze sequence data.

iGenomics can be used in virtually any location because of the inherent portability of mobile devices like the iPad and iPhone. iGenomics implements the same advanced bioinformatics algorithms that are used for rapid alignment and analysis for other platforms. Consequently, the true novelty of this application is not in the algorithms used, but rather how they have been implemented in a mobile environment. The entire workflow for iGenomics is designed to be very simple and intuitive. A user effortlessly picks a reads file to analyze and, once selected, the alignment, variant calling, and visualization are completed within seconds. This is accomplished without any internet connectivity through an optimized implementation in Objective-C.

iGenomics is designed for quickly computing detailed genetic information about specific mutations within different viral or bacterial genomes. An important use case of iGenomics could be a researcher with limited computational resources sequencing cDNA of a coronavirus sample, loading and aligning the cDNA reads with iGenomics, and getting a first analysis of the coronavirus mutations within a few seconds. To support this capability, we have developed a tutorial with the MinION reads (SRX7615629) and consensus genome (MN938384.1) from patient HKU-SZ-002a, as well as the consensus genome from a bat SARS-like coronavirus isolate (MG772934.1/) previously used for comparisons (Chan et al. 2020) (http://schatz-lab.org/iGenomics/). Following the tutorial, these data can easily be downloaded on one's iOS device and imported directly into iGenomics to be analyzed. Another promising capability of iGenomics is its ability to load reference genomes and reads from outside sources, perform alignment and variant calling, and export the results all without any internet access. For example, by using Airdrop to both import and export data from iGenomics, a researcher can analyze DNA in remote locations without any internet connectivity. As the MinION uses a USB connection that is not available on an iPhone or iPad, users will first need to collect the raw sequencing data on their laptop or server as well as use these platforms to base call the signal data into nucleotide sequences. However, once sequencers are available that can read DNA directly into iOS devices, iGenomics will work out of the box to allow for importing of this sequenced data, eliminating the requirement for a laptop in the end-to-end analysis pipeline.

Future developments for iGenomics are far reaching as DNA sequencing instruments continue to evolve to the point where they could be directly attached or integrated with mobile devices. In fact, Oxford Nanopore has announced that they hope to have a new sequencer, named the "SmidgION", that connects directly to iOS devices available for researchers in the near future

(https://nanoporetech.com/products/smidgion). At that point, using mobile sequencing technology with iGenomics, DNA can truly be sequenced, aligned, and analyzed anywhere and absolute mobility of the genomics field will be achieved. As the processing power and memory contained within mobile devices improves, so will the overall performance of iGenomics in handling even larger and more complex samples.

Methods

The implementation of iGenomics follows the state-of-the-art algorithms and data structures used in standard bioinformatics applications. However, the visualization of the read alignments and mutations is unique to iGenomics and was created with the intention of allowing the user to have powerful analysis capabilities while still maintaining a simplistic mobile-friendly interface.

1. Indexing the genome with the Burrows-Wheeler Transform (BWT)

The Burrows-Wheeler Transform (BWT) is constructed by lexicographically sorting the cyclic permutations of the input genome appended by a end-of-string character. By convention, we use a dollar sign ('$') as the end-of-string character, which has a lexicographical value less than any letter in the English alphabet and ensures the end of the original sequence can be found. For example, the cyclic permutations of the string "CAT" with the end-of-string character "$" are: "CAT$", "AT$C", "T$CA", and "$CAT", which can be sorted as "$CAT", "AT$C", CAT$" and "T$CA". This sorted list creates what is known as the Burrows-Wheeler Matrix (BWM). Then, to compute the BWT from the sorted permutations, the last character of each row in the matrix is extracted in order and appended to a string **(Figure 5)**.

To first lexicographically sort the cyclic permutations, a quick and efficient sorting algorithm must be used so that this function is fully optimized. iGenomics uses a version of QuickSort, a divide-and-conquer sorting algorithm, because on average it takes O(n log n) time for n objects to be sorted. Although there are now some more efficient BWT construction algorithms (Belazzougui et al. 2020), given iGenomics is targeted towards relatively small genomes (<100,000bp), the amount of time for BWT sorting is negligible compared to the time to align the reads. Finally, to obtain the BWT from the sorted array, the final character of each row in the matrix is copied into a string with the first character copied having the first position, the second character copied having the second position, and so forth.

2. Read alignment

iGenomics uses a seed-and-extend process for read alignment in which first relatively short exact matches, known as seeds, are found using the BWT, after which they are then extended into end-to-end alignments using dynamic programming. The seed size is based upon the maximum edit distance (a user-specified parameter) allowed for a read that successfully aligns to be considered a match. The maximum edit distance is inputted as a decimal value edit rate, and multiplying that value by the length of the given read will give the maximum possible edit distance we allow when aligning that read. During the aligning process, each read is split into the edit distance plus one segment of equal length. This relies on the widely used technique that if the string matches with at most X edits, then at least 1/(X+1) of the segments must still match without error (Baeza-Yates and Perleberg 1996). For example, if the user allows only 1 edit, the algorithm divides the read into left and right halves (1/(1+1)) knowing that the correct alignment will include an exact match of one of those segments.

Exact matching means finding all of the places in the reference genome where a given query matches exactly, character-for-character across its entire length (Langmead, 2012). To do this

effectively, the trait of the BWT known as the Last-First Property is used as the basis for an exact matching algorithm. The Last-First property states that the occurrence of any character in the last column of the BWM, which is the BWT, corresponds to the same occurrence of that character in the first column of the BWM. Using the first column of the BWM and the BWT to create an FM-index, the algorithm navigates the rows of the index which contain exact matches and then converts these positions from the BWT to positions in the reference genome **(Figure 6)**.

After the seeds are found, iGenomics computes the end-to-end edit distance allowing for substitutions as well as insertions and deletions (Smith and Waterman 1981) **(Figure 7)**. To make this as efficient as possible, iGenomics uses a banded computation. This method works by only computing a subset of the dynamic programming matrix, a band of the edit distance table, with the band having a standard width of (the maximum edit distance * 2 + 1). To determine where to begin the band computation, iGenomics attempts to exact match a 20bp substring of the read. A substring length of 20bp was chosen as we found that represented the optimal tradeoff in terms of performance and reliability of identifying alignments. If the exact match is successful, the banded distance will be computed relative to the matched position of the substring. If the exact match is unsuccessful, an exact match with the 20bp substring of the read starting at the second character will be attempted. This process continues with the substrings continuously moving one character over until either the read successfully aligns or none of the exact matched 20bp substrings yields a successful alignment.

3. Coverage profile and variant identification

The coverage profile concisely summarizes how the reads are aligned to the genome (**Figure 8**). The internal data structure for the profile is a coverage profile matrix, which spans the genome and at each position contains a row for the number of: matched base-pairs, A, C, G, T, and (non-base-pair) deletion characters. The matched positions of each read are tallied and the characters of the read are added, so that the positions of the matrix that the read overlaps are marked within the matrix. Once the coverage profile matrix is completely generated, variants can be identified, a graphical representation of the profile can be formed, and the number of alignments can easily be seen.

Variants are identified by scanning the array of matched characters, and at each position if the matched character differs from the reference character, a mutation, or variant, would be reported (Li et al. 2009). The major challenge of this analysis is distinguishing sequencing errors from real mutations, and differentiating between homozygous and heterozygous mutations. In a diploid genome, homozygous mutations are mutations that occur on both copies of a chromosome whereas heterozygous mutations occur on one copy of a chromosome but not both. iGenomics recognizes heterozygous mutations as positions in the genome where there is a nearly equal coverage of more than one base existing in the set of aligned reads according to a user-specified relative minimum heterozygosity threshold. Thus, if two or more bases at a position have relative coverages greater than that threshold, the mutation present at that position is considered to be heterozygous. In haploid species, such as the viral and bacterial pathogens described above, this threshold is used to find variants that occur within a minimum allele frequency within the population.

Immediately after alignment has completed, each position within the reference genome is assigned a value indicating whether the reads at that position matched either exactly, heterozygously, homozygously, heterozygously where there is a known mutation, or homozygously where there is a known mutation. This allows iGenomics to highlight all mutations with their associated heterozygosity and importance. Known mutations are loaded through a user-inputted text file. This file contains each known (important) mutation's reference base, mutated base, position, segment (or chromosome) the mutation is expected to occur in, and a free-text description of what this mutation indicates. The known mutations functionality enables iGenomics to be specifically targeted for the analysis and treatment of different genomes, such as known mutations associated with Influenza

antiviral resistance.

4. Visualizations and interactive analysis

The main challenge with the Graphical User Interface (GUI) was to create one that was both useful and unique when compared to other desktop DNA analysis software. The key to achieving these goals was to take advantage of the distinctive features of the iOS environment. Ultimately, a custom graphics engine was built to handle the constant redrawing of the analysis interface and, visually, this engine sits on top of Apple's CoreGraphics library. In addition to the analysis interface, a utility interface was developed, which contains features for rapidly analyzing and quickly navigating the alignments.

The solution to developing this interactive analysis screen was to employ many touch-related functions that are natural to anyone who has ever used a touch screen mobile device (**Supplemental Figures 2-8**). Scrolling requires a simple finger drag while viewing a large-scale version of the coverage profile merely requires performing a pinch gesture on the screen. The information pertaining to mutations can be viewed at any position by tapping on one of the reference genomes or found genome boxes at that position. Even this action takes advantage of the mobile iOS environment because a popover view is used to display the information at the tapped position. At the bottom of the screen, there is a variable scrubbing speed slider so that the user can move across the genome quickly or at a slower rate by dragging up while moving the slider.

Simple functions such as searching for a specific query or position are also included in the analysis view. To minimize clutter on the screen, when a user searches for a certain string, he/she is instantly taken to the next occurrence of that string, as opposed to displaying a large list of positions to the user. One of the most notable of these functions is the ability to change the minimum relative heterozygosity value (known as mutation coverage within iGenomics) on the fly through a slider. Once the user has concluded analyzing on the mobile device, he/she has the option to export mutations and analysis data via a variety of means: email, Dropbox, Airdrop, or sharing via installed apps (such as Google Drive). The mutations are outputted in a VCF (Variant Call Format) file format so that they are compatible with traditional desktop analysis software.

5. Flu Isolate Sequencing

Sample collection and amplification. Clinical specimens of nasopharyngeal swabs were collected from patients in New York City in the 2014-2015 flu season as previously described (Ding et al. 2019). The specimen used in this study was designated as A/New York/A39/2015 (H3N2) and is available in the SRA as sample ID SAMN08454624. Briefly, the RNA was eluted in 30 µl of RNase-free water and 3 µl was used as a template for the amplification of the entire influenza A or B genome using previously described Multi-segment RT-PCR (M-RTPCR) method (Zhou et al. 2009). The presence of the cDNA copies of the genomic segments were examined by running 3 µl of the M-RTPCR amplicons on a 0.8% agarose electrophoresis gel. The influenza genomic amplicons were purified using a 1x Agencourt AMPure XP purification step and assessed by Qubit analysis to quantify the mass of the double-stranded cDNA present.

Nanopore MinION sequencing. The library preparation and sequencing procedures were performed following manufacturer's instructions for the Nanopore Sequencing using the SQK-MAP006 kit. Purified DNA was used for end repair and dA-tailing, followed by 1x AMPure XP beads purification. The resultant DNA was quantitated by Qubit analysis and the molarity was further determined by using Agilent 2200 TapeStation system with a Genomic DNA ScreenTape. Next, 0.2 pmoles of the DNA was used in adaptor ligation, and the reaction was purified using MyOne

C1-beads. The final DNA was eluted in 25 µl Elution Buffer and is called Pre-sequencing Mix. For the SQK-MAP006 sequencing kit, 12 µl Pre-sequencing Mix was combined with 75 µl 2x Running Buffer, 59 µl nuclease-free water, and 4 µl Fuel Mix and then loaded into the FLO-MAP003 flow cell. A re-loading was also performed. The sequencing was run on the MIN-MAP001 MinION sequencing device, which was control by the MinKNOW software using the MAP_48Hr_Sequencing_Run.py script provided by Oxford Nanopore or using the MAP_140to5xVoltage_Tuned_plus_Yield_Sequencing_Run.py script provided by John Tyson. Raw data was uploaded to the cloud-based Metrichor platform and basecalling was performed using the application of 2D Basecalling for SQK-MAP005 Rev 1.62 or 2D Basecalling for SQK-MAP006 Rev 1.62.

Illumina MiSeq sequencing. The sample was prepared for sequencing on the Illumina MiSeq platform according to the manufacturer's protocol (15039740 v01) as previously described (Ding et al. 2019). Sequencing data was then generated by a 2x300bp run using an Illumina MiSeq 600 Cycle v3 reagent kit.

Data Availability

All sequencing data (genuine and simulated) along with a tutorial on iGenomics are available online: http://schatz-lab.org/iGenomics/.

Acknowledgements

We would like to thank Jaak Raudsepp for his helpful discussions and involvement during the development of iGenomics. We would also like to thank Dr. Mirella Salvatore for providing the flu samples. The project was supported in part by the US National Science Foundation award (DBI-1350041) to MCS. This paper is dedicated to Albert Palatnick, grandfather of Aspyn Palatnick, who has fueled Aspyn's fascination with science, technology, and innovation since he was a child. MCS has received travel funding from Oxford Nanopore Technologies Limited.

References

Baeza-Yates, Ricardo A., and Chris H. Perleberg. 1996. “Fast and Practical Approximate String Matching.” *Information Processing Letters* 59 (1): 21–27.

Belazzougui, Djamal, Fabio Cunial, Juha Kärkkäinen, and Veli Mäkinen. 2020. “Linear-Time String Indexing and Analysis in Small Space.” *ACM Transactions on Algorithms*, 17, 16 (2): 1–54.

Burrows, Michael, and David Wheeler. 1994. “A Block-Sorting Lossless Data Compression Algorithm.” In *DIGITAL SRC RESEARCH REPORT*. http://citeseerx.ist.psu.edu/viewdoc/summary?doi=10.1.1.37.6774.

Castro-Wallace, Sarah L., Charles Y. Chiu, Kristen K. John, Sarah E. Stahl, Kathleen H. Rubins, Alexa B. R. McIntyre, Jason P. Dworkin, et al. 2017. “Nanopore DNA Sequencing and Genome Assembly on the International Space Station.” *Scientific Reports* 7 (1): 18022.

Chan, Jasper Fuk-Woo, Shuofeng Yuan, Kin-Hang Kok, Kelvin Kai-Wang To, Hin Chu, Jin Yang, Fanfan Xing, et al. 2020. “A Familial Cluster of Pneumonia Associated with the 2019 Novel Coronavirus Indicating Person-to-Person Transmission: A Study of a Family Cluster.” *The Lancet*, January. https://doi.org/10.1016/S0140-6736(20)30154-9.

Ding, Tao, Timothy Song, Bin Zhou, Adam Geber, Yixuan Ma, Lingdi Zhang, Michelle Volk, et al. 2019. “Microbial Composition of the Human Nasopharynx Varies According to Influenza Virus Type and Vaccination Status.” *mBio* 10 (4). https://doi.org/10.1128/mBio.01296-19.

Faria, Nuno Rodrigues, Ester C. Sabino, Marcio R. T. Nunes, Luiz Carlos Junior Alcantara, Nicholas J. Loman, and Oliver G. Pybus. 2016. “Mobile Real-Time Surveillance of Zika Virus in Brazil.” *Genome Medicine* 8 (1): 97.

Ferragina, P., and G. Manzini. 2000. “Opportunistic Data Structures with Applications.” In *Proceedings 41st Annual Symposium on Foundations of Computer Science*, 390–98.

Giovanetti, Marta, Nuno Rodrigues Faria, José Lourenço, Jaqueline Goes de Jesus, Joilson Xavier, Ingra Morales Claro, Moritz U. G. Kraemer, et al. 2020. “Genomic and Epidemiological Surveillance of Zika Virus in the Amazon Region.” *Cell Reports* 30 (7): 2275–83.e7.

Goodwin, Sara, John D. McPherson, and W. Richard McCombie. 2016. “Coming of Age: Ten Years of next-Generation Sequencing Technologies.” *Nature Reviews. Genetics* 17 (6): 333–51.

Hussain, Mazhar, Henry D. Galvin, Tatt Y. Haw, Ashley N. Nutsford, and Matloob Husain. 2017. “Drug Resistance in Influenza A Virus: The Epidemiology and Management.” *Infection and Drug Resistance* 10 (April): 121–34.

Krammer, Florian, Gavin J. D. Smith, Ron A. M. Fouchier, Malik Peiris, Katherine Kedzierska, Peter C. Doherty, Peter Palese, et al. 2018. “Influenza.” *Nature Reviews. Disease Primers* 4 (1): 3.

Kurtz, Stefan, Adam Phillippy, Arthur L. Delcher, Michael Smoot, Martin Shumway, Corina Antonescu, and Steven L. Salzberg. 2004. “Versatile and Open Software for Comparing Large Genomes.” *Genome Biology* 5 (2): R12.

Langmead, Ben, Cole Trapnell, Mihai Pop, and Steven L. Salzberg. 2009. “Ultrafast and Memory-Efficient Alignment of Short DNA Sequences to the Human Genome.” *Genome Biology* 10 (3): R25.

Li, Heng. 2013. “Aligning Sequence Reads, Clone Sequences and Assembly Contigs with BWA-MEM.” *arXiv:1303.3997 [q-bio.GN]*. arXiv. http://arxiv.org/abs/1303.3997.

———. 2018. “Minimap2: Pairwise Alignment for Nucleotide Sequences.” *Bioinformatics* 34 (18): 3094–3100.

Li, Heng, Bob Handsaker, Alec Wysoker, Tim Fennell, Jue Ruan, Nils Homer, Gabor Marth, Goncalo Abecasis, Richard Durbin, and 1000 Genome Project Data Processing Subgroup. 2009. “The Sequence Alignment/Map Format and SAMtools.” *Bioinformatics* 25 (16): 2078–79.

McKenna, Aaron, Matthew Hanna, Eric Banks, Andrey Sivachenko, Kristian Cibulskis, Andrew Kernytsky, Kiran Garimella, et al. 2010. “The Genome Analysis Toolkit: A MapReduce Framework for Analyzing next-Generation DNA Sequencing Data.” *Genome Research* 20 (9):

1297–1303.
Oude Munnink, Bas B., David F. Nieuwenhuijse, Mart Stein, Áine O'Toole, Manon Haverkate, Madelief Mollers, Sandra K. Kamga, et al. 2020. "Rapid SARS-CoV-2 Whole-Genome Sequencing and Analysis for Informed Public Health Decision-Making in the Netherlands." *Nature Medicine* 26 (9): 1405–10.
Payne, Alexander, Nadine Holmes, Vardhman Rakyan, and Matthew Loose. 2019. "BulkVis: A Graphical Viewer for Oxford Nanopore Bulk FAST5 Files." *Bioinformatics* 35 (13): 2193–98.
Pomerantz, Aaron, Nicolás Peñafiel, Alejandro Arteaga, Lucas Bustamante, Frank Pichardo, Luis A. Coloma, César L. Barrio-Amorós, David Salazar-Valenzuela, and Stefan Prost. 2018. "Real-Time DNA Barcoding in a Rainforest Using Nanopore Sequencing: Opportunities for Rapid Biodiversity Assessments and Local Capacity Building." *GigaScience* 7 (4). https://doi.org/10.1093/gigascience/giy033.
Quick, Joshua, Nicholas J. Loman, Sophie Duraffour, Jared T. Simpson, Ettore Severi, Lauren Cowley, Joseph Akoi Bore, et al. 2016. "Real-Time, Portable Genome Sequencing for Ebola Surveillance." *Nature* 530 (7589): 228–32.
Robinson, James T., Helga Thorvaldsdóttir, Wendy Winckler, Mitchell Guttman, Eric S. Lander, Gad Getz, and Jill P. Mesirov. 2011. "Integrative Genomics Viewer." *Nature Biotechnology* 29 (1): 24–26.
Shendure, Jay, Shankar Balasubramanian, George M. Church, Walter Gilbert, Jane Rogers, Jeffery A. Schloss, and Robert H. Waterston. 2017. "DNA Sequencing at 40: Past, Present and Future." *Nature* 550 (7676): 345–53.
Smith, T. F., and M. S. Waterman. 1981. "Identification of Common Molecular Subsequences." *Journal of Molecular Biology* 147 (1): 195–97.
Thielen, Peter M., Shirlee Wohl, Thomas Mehoke, Srividya Ramakrishnan, Melanie Kirsche, Oluwaseun Falade-Nwulia, Nidia S. Trovao, et al. 2020. "Genomic Diversity of SARS-CoV-2 During Early Introduction into the United States National Capital Region." *medRxiv : The Preprint Server for Health Sciences*, August. https://doi.org/10.1101/2020.08.13.20174136.
Viehweger, Adrian, Sebastian Krautwurst, Kevin Lamkiewicz, Ramakanth Madhugiri, John Ziebuhr, Martin Hölzer, and Manja Marz. 2019. "Direct RNA Nanopore Sequencing of Full-Length Coronavirus Genomes Provides Novel Insights into Structural Variants and Enables Modification Analysis." *Genome Research* 29 (9): 1545–54.
Wick, Ryan R., Louise M. Judd, and Kathryn E. Holt. 2019. "Performance of Neural Network Basecalling Tools for Oxford Nanopore Sequencing." *Genome Biology* 20 (1): 129.
Zhou, Bin, Matthew E. Donnelly, Derek T. Scholes, Kirsten St George, Masato Hatta, Yoshihiro Kawaoka, and David E. Wentworth. 2009. "Single-Reaction Genomic Amplification Accelerates Sequencing and Vaccine Production for Classical and Swine Origin Human Influenza a Viruses." *Journal of Virology* 83 (19): 10309–13.

Figure Captions

Figure 1. iGenomics iPhone screenshots **(top-left)** Alignments display; **(top-right)** Alignment display zoomed-out; **(middle-left)** Coverage profile; **(middle-right)** Coverage profile zoomed-out, **(bottom)** Known mutations display. In the known mutations display, green indicates the mutation is not present, dark red indicates the listed mutation is present and the mutation is homozygous, and pink indicates the listed mutation is present and the mutation is heterozygous. In both the alignments display and coverage profile, there is an indicator in the top right of the form [X, Y] that represents the minimum coverage X across all positions and maximum coverage Y across all positions.

Figure 2. Runtimes for simulated reads from five reference genomes. The data sets consisted of reads averaging 100x coverage and a reference file. Each data set was tested, defined as aligning then variant calling, using iGenomics running on an iPhone and a BWA/Samtools pipeline running on a laptop. The technical specifications of the iPhone and laptop used for testing are described in the Results section. Each trend line indicates the runtime for each data set using the denoted alignment and analysis software- iG for iGenomics and bwa for the BWA/Samtools pipeline. The dotted lines indicate the specific measurements recorded.

Figure 3. Mutation identification accuracy for simulated H1N1 flu datasets of varying mutation rates and error rates for iGenomics (left) and the BWA-MEM/Samtools (right) pipeline. The top, middle, and bottom plots show recall, precision, and F-score, respectively.

Figure 4. iGenomics runtime vs. BWA/Samtools pipeline runtime for simulated datasets of constant mutation rates and sequence error rates of H1N1 for varying read lengths.

Figure 5. Diagram of how the Burrows-Wheeler Transform is created. (left) All cyclic permutations of the text “GATTACA”. (right) The Burrows-Wheeler Matrix of the text consisting of the sorted cyclic permutations of the text.

Figure 6. A diagram showing the exact match algorithm by repeated application of the Last-First property using the characters of the query string.

Figure 7. A diagram showing how edit distance is computed for two strings. Each cell of the matrix represents the minimum of three possible values: 1) the left cell plus one (representing the cost of adding a gap on the left string); 2) the upper cell plus one (representing the cost of adding a gap on the top string; and 3) the upper left cell plus zero, if the top string equals the left string, or one, if the characters do not match to account for the cost of another substitution.

Figure 8. A table showing how the coverage profile is represented within iGenomics, summarizing how the reads align to the reference genome (an example of reads aligned to a reference genome is shown in Figure 1). As can be seen in the 6th column, there is a mutation where the base C was found when the reference was base G.

Table Captions

Table 1. Comparison between iGenomics and BWA-MEM/Samtools pipeline for real reference genomes and reads obtained from MinION (Nanopore) and MiSeq sequencers.

Table 2. Table indicating alignment details for simulated datasets aligned using iGenomics to a pan-genome composed of multiple Influenza genomes. The pH1N1 reads were simulated from the H1N1pdm09 (A/California/04/2009) genome and the H3N2 reads were simulated from the H3N2 (A/NewYork/03/2015) genome.

Table 1

	MinION Ebola Data	
	**iGenomics***	**BWA+SAMtools**
**Alignment Rate**	99.24%	100.00%
**Runtime**	24.71s	428.96s
**Precision, Recall, Accuracy (when compared to SAM[illegible]**	61.54%, 66.67%, [illegible]	N/A
**Precision, Recall, Accuracy (when compared to [illegible]**	N/A	N/A

*Unreported heterozygosity is present in the mutations called.

⁺This method of variant calling is considered to be the ground-truth. BWA+SA[illegible]

	MinION H3N2 Data	
	**iGenomics**	**BWA+SAMtools**
**Alignment Rate**	99.36%	98.08%
**Runtime**	28.04s	180.49s
**Precision, Recall, Accuracy (when compared to SAM[illegible]**	40.24%, 93.44%, [illegible]	N/A
**Precision, Recall, Accuracy (when compared to SAM[illegible]**	74.82%, 87.12%, [illegible]	99.45%, 49.80%, [illegible]

⁺This method of variant calling is considered to be the ground-truth.

MinION Zika Data	
**iGenomics***	**BWA+SAMtools**
81.46%	94.11%
13.11s	189.19s
86.79%, 79.77%, 88.18%	N/A
86.16%, 88.96%, 87.54%	83.24%, 93.51%, 88.97%

Mtools has an N/A (Not Applicable) in these

MiSeq H3N2 Data	
**iGenomics**	**BWA+SAMtools**
98.18%	98.58%
4.78s	27.59s
N/A	N/A
99.73%, 99.73%, 99.72%	N/A

Click here to access/download;Table;Table 2.xlsx

Table 2

	MinION Simulated Data	
	**pH1N1**	**H3N2**
**Alignment Rate**	100.00%	100.00%
**Runtime**	< 4.25s	< 4.17s
**Segment Identification Rate**[+]	> 99.11%	> 95.04%

[+]Segment identification rate is the number of alignments that align[illegible]

Illumina Simulated Data	
**pH1N1**	**H3N2**
100.00%	> 99.84%
< 1.60s	< 1.63s
> 99.84%	> 93.02%

ed to the correct reference

Figure 1

Click here to access/download;Figure;Figure 1. iGenomics Interface.pdf

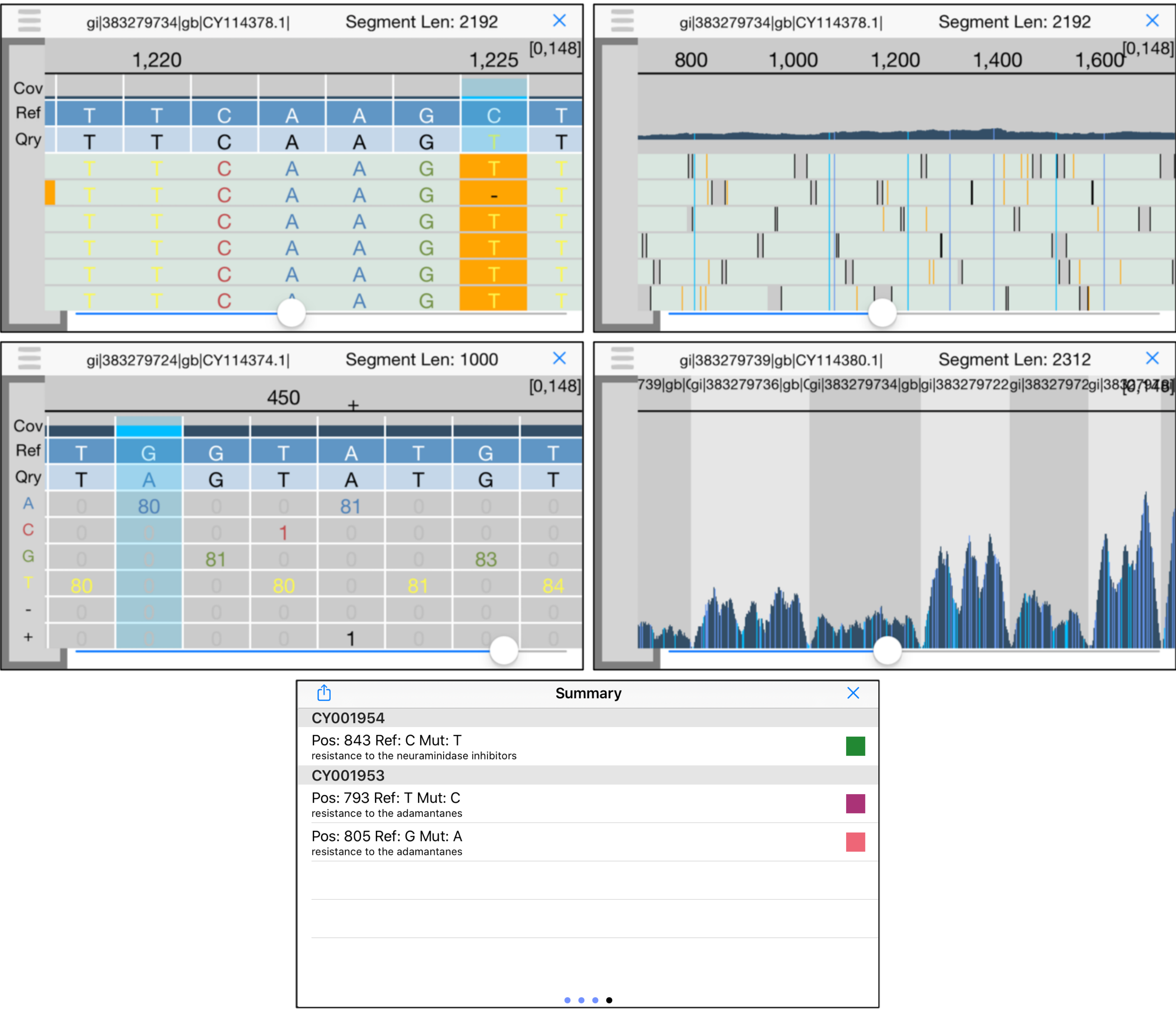

Figure 2

Click here to access/download;Figure;Figure 2. Runtime vs Reference Length.pdf

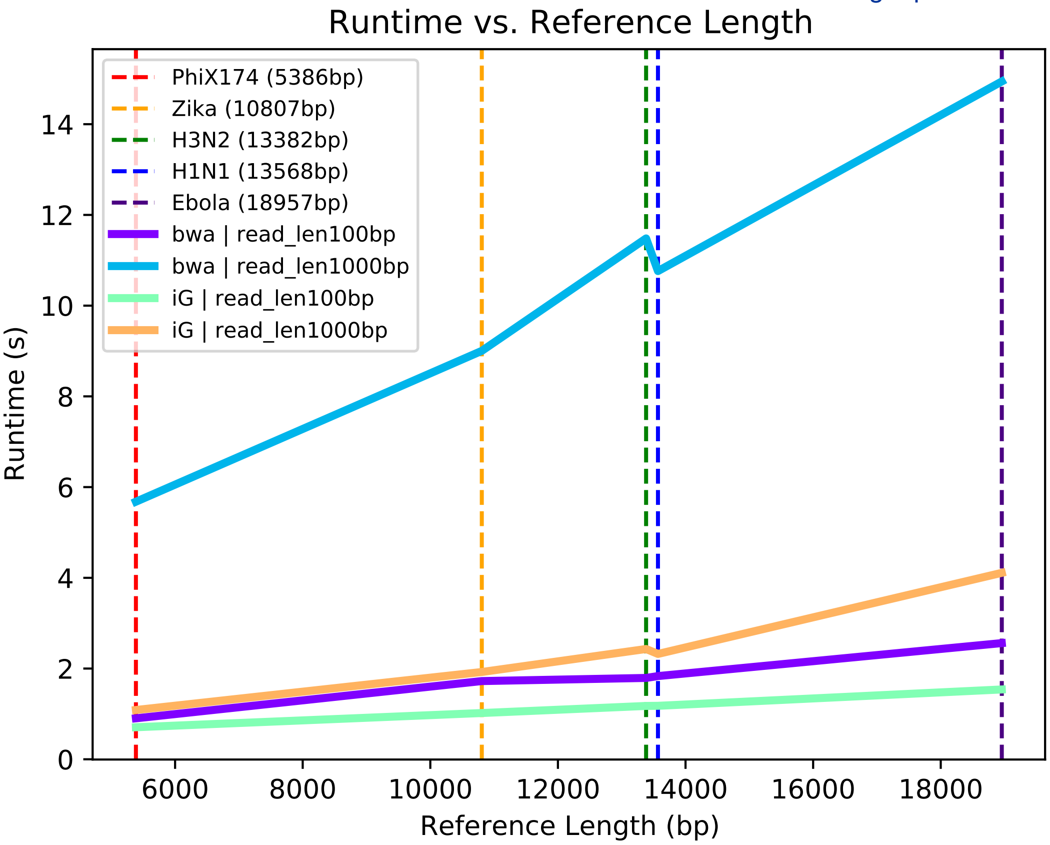

Figure 3

Click here to access/download;Figure;Figure 3. Variant Accuracy.png

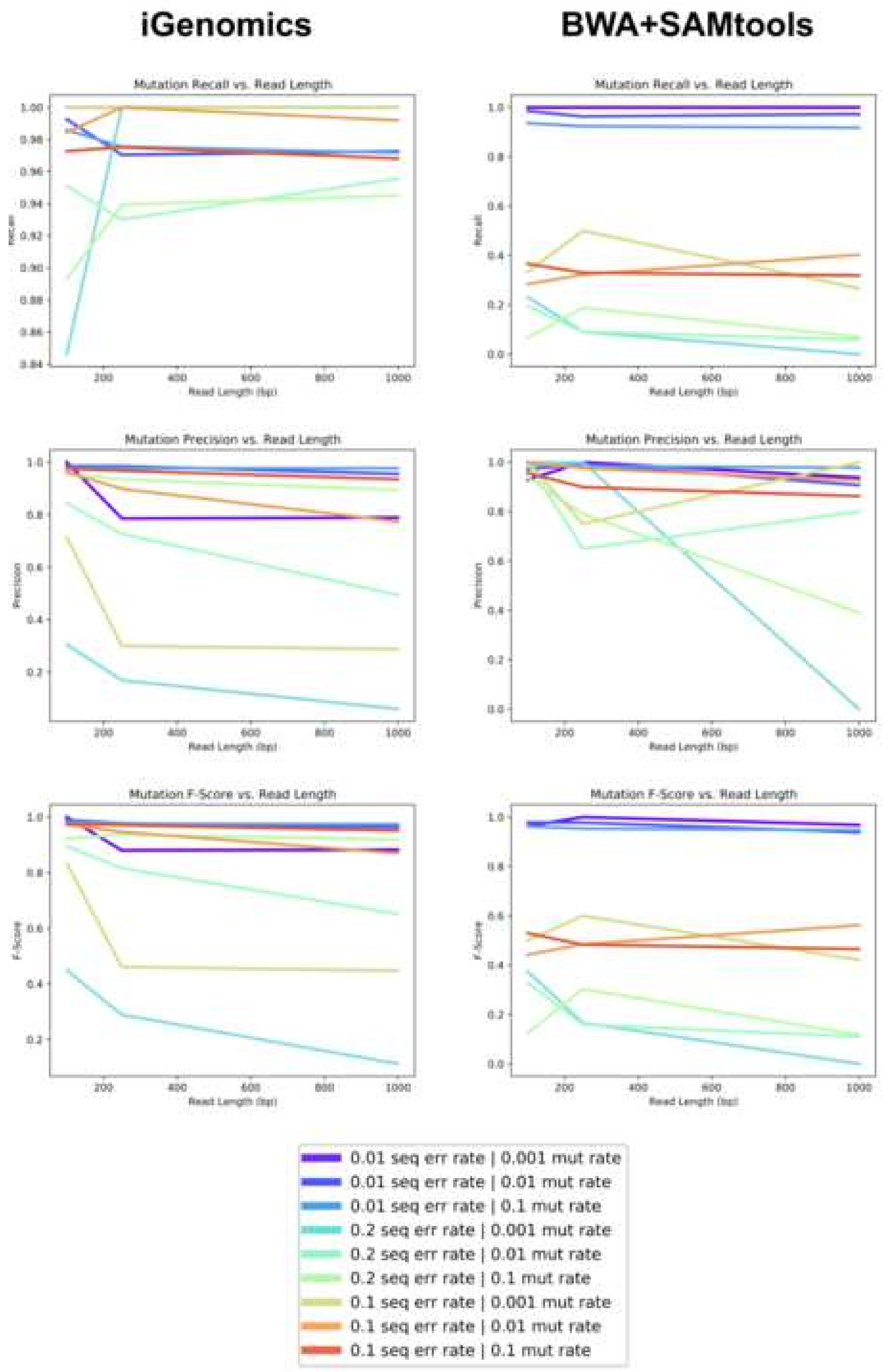

Figure 4

Click here to access/download;Figure;Figure 4. iGenomics vs BWA Runtime.pdf

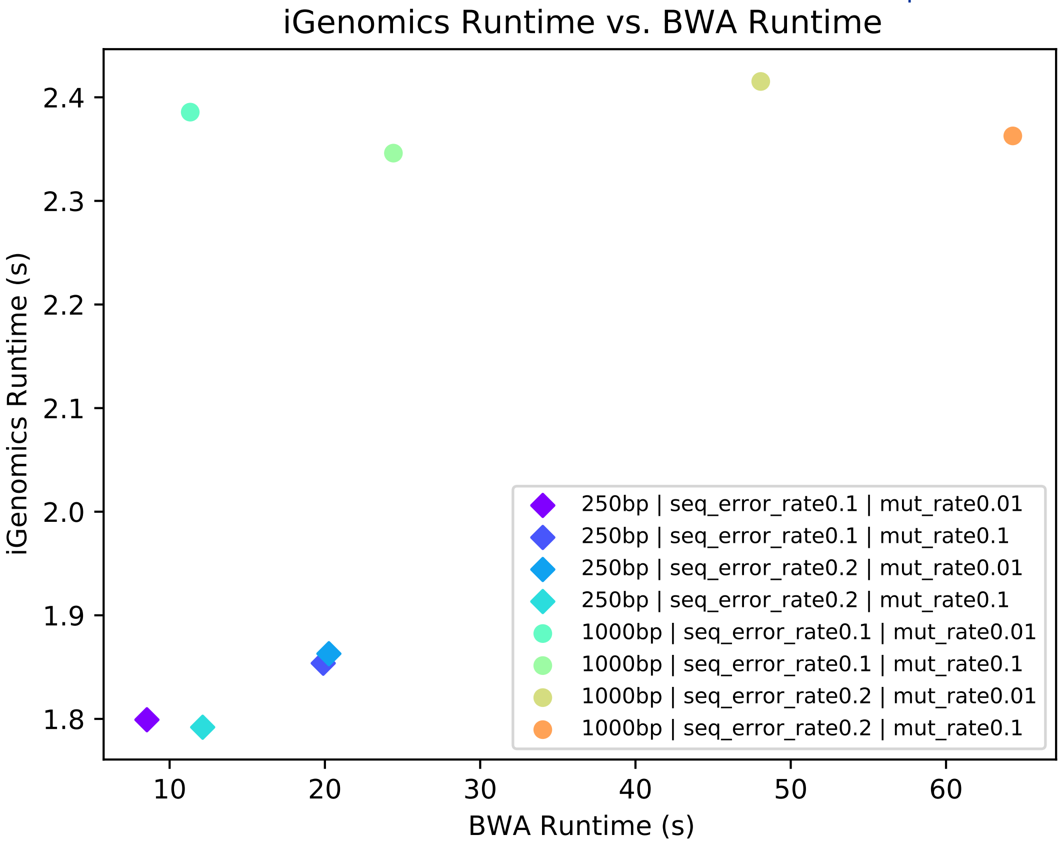

Figure 5

Click here to access/download;Figure;Figure 5. BWT Construction.png

Creating the BWT for GATTACA

Unsorted Cyclic Permutations of GATTACA

G	A	T	T	A	C	A	$
A	T	T	A	C	A	$	G
T	T	A	C	A	$	G	A
T	A	C	A	$	G	A	T
A	C	A	$	G	A	T	T
C	A	$	G	A	T	T	A
A	$	G	A	T	T	A	C
$	G	A	T	T	A	C	A

Sorted Cyclic Permutations of GATTACA

$	G	A	T	T	A	C	A
A	$	G	A	T	T	A	C
A	C	A	$	G	A	T	T
A	T	T	A	C	A	$	G
C	A	$	G	A	T	T	A
G	A	T	T	A	C	A	$
T	A	C	A	$	G	A	T
T	T	A	C	A	$	G	A

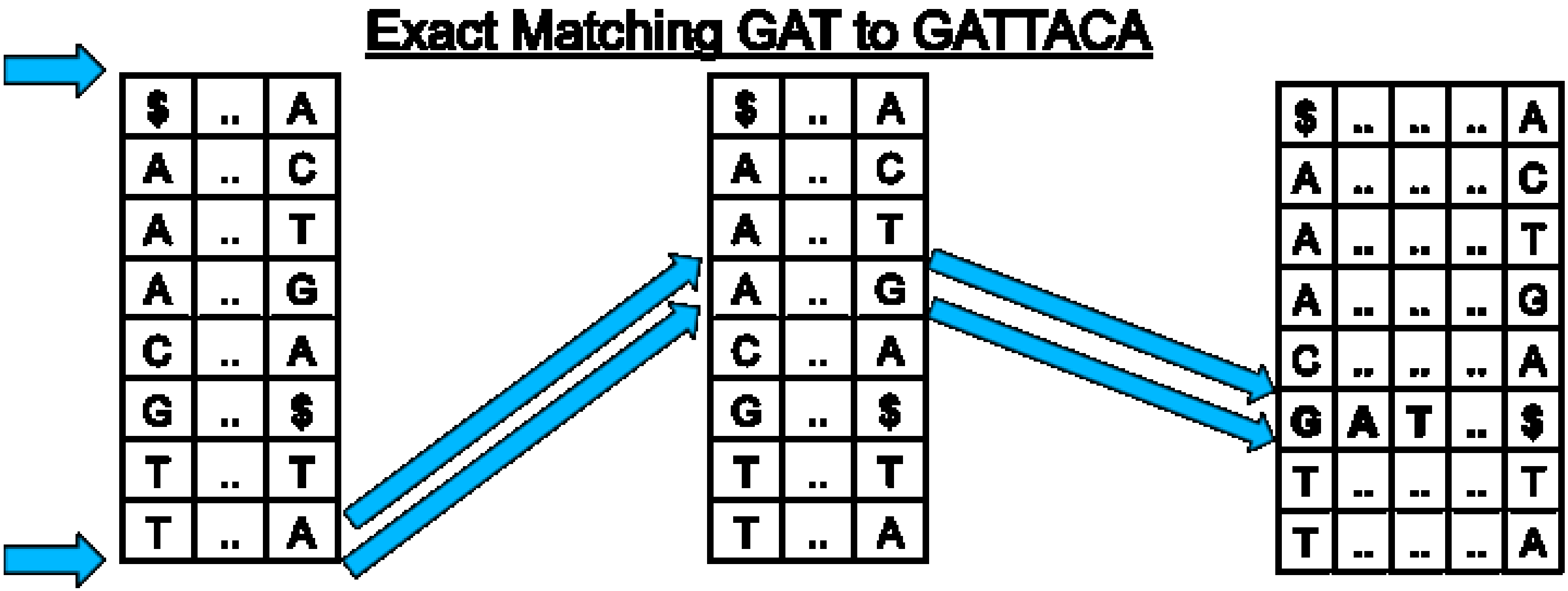
Exact Matching GAT to GATTACA
$ .. A
A .. C
A .. T
A .. G
C .. A
G .. $
T .. T
T .. A
$ .. .. .. A
A C
A .. .. .. T
A .. .. .. G
C .. .. .. A
G A T .. $
T .. .. .. T
T .. .. .. A

Figure 7

Click here to access/download;Figure;FIgure 7. Edit Distance.png

Computing Edit Distance for GATTTACA to GATTACA

		G	A	T	T	T	A	C	A
	0	1	2	3	4	5	6	7	8
G	1	0	1	2	3	4	5	6	7
A	2	1	0	1	2	3	4	5	6
T	3	2	1	0	1	2	3	4	5
T	4	3	2	1	0	1	2	3	4
A	5	4	3	2	1	1	1	2	3
C	6	5	4	3	2	2	2	1	2
A	7	6	5	4	3	3	3	2	1

GATTTACA
GATT-ACA

The final edit distance

 Click here to access/download;Figure;Figure 8. Coverage Profile.png

Reference	G	A	A	T	G	G	C
Found	G	A	A	T	G	C	C
A	0	3	3	0	3	0	0
C	0	0	0	0	0	3	3
G	3	0	0	0	0	0	0
T	0	0	0	3	0	0	0

Supplementary Figures

Click here to access/download
Supplementary Material
Supplemental Figures.pdf

To the editor,

Thank you and the reviewers for your detailed and insightful comments. We have addressed all of the concerns in the revised manuscript. We have also remade many of the figures to improve their readability, and have included high resolution versions as separate file uploads. Please see our point-by-point response below with the reviewer comments in blue and our responses in black text. Our changes to the manuscript are also highlighted in yellow.

Thank you,

Michael Schatz (on behalf of all of the authors)

~~~~~~

Reviewer 1

Palatnick and colleagues present iGenomics, a mobile-device based DNA data anlysis software. Having worked on portable sequencing for many years, I am very excited by this software, and I am happy to see this paper published in GigaScience after some revisions.

Thank you for your support!

I think that the tone of the paper needs to be turned down a bit. I really enjoyed using the tool and think that it is a great first step into a new direction, but being novel, there are still issues and limitations that weren't touched on in the manuscript. Furthermore, it reads a bit more like an advertisement in some parts. Please, refrain from overselling portable sequencing and your tool. I think that simply stating the status-quo and what might be possible in the future - with iGenomics being a first step - is exciting enough!

Here are some suggestions on how to improve the current draft:

1) The paper lacks a discussion of the current literature and applications of portable sequencing for viral and bacterial sequencing analyses - which helps to set the context. There is a lot of exciting research being conducted in this area and I think mentioning some real world applications and why these benefit from portable technologies will be very helpful and interesting to the readers.

Thank you for this suggestion. We have expanded the introduction to highlight some of these recent applications as follows:

"Because of its small size, Nanopore sequencing has been used in several environments that would be unthinkable for alternative instruments as diverse as monitoring the Ebola outbreaks
~~~~~~

in remote areas of Africa (Quick et al. 2016), monitoring Zika outbreaks in South America (Giovanetti et al. 2020), exploring reptile specimens in the rainforest (Pomerantz et al. 2018), and even on the International Space Station (Castro-Wallace et al. 2017). Nanopore sequencing has also played an important role in monitoring the transmission of SARS-COVID-19 around the world (Viehweger et al. 2019; Oude Munnink et al. 2020; Thielen et al. 2020).”

2) The authors haven’t touched on issues, which limit the use of iGenomics a bit at the moment. I really think this is an interesting step in the right direction, but e.g. since basecalling will need to be performed on a laptop/server anyway, I think the use for a phone-based software is limited. Please, discuss this issue and possible future solutions. This current limitation does not limit the excitement about the future of mobile-phone-based DNA data analysis.

As with you, we think there are many exciting and important applications for mobile sequence analysis even without integrated basecalling, especially to broaden the community of potential researchers and citizen scientists that may be interested to perform some analysis but lack any formal training. We have expanded on these points in the introduction (all see Reviewer 1 comment 7 below).

Unfortunately, the document I had for review didn’t have line numbers, so I copy and pasted the sentences into this doc to make it easy to follow.

I hope my comments are useful and I am excited to see more of the tool in the future!

Sincerely, Stefan Prost, PhD

Detailed Comments:

Abstract

1. “Leading this trend is the Oxford Nanopore sequencing platform, which currently offers the hand-held MinION instrument and even smaller instruments on the near horizon.” should read: “Leading this trend is Oxford Nanopore Technologies, which currently offers the hand-held MinION instrument and even smaller instruments may be on the ~~near~~ horizon.” They have advertised the SmidgION ever since I started using ONT 5-6 years ago. I don’t think it really is anywhere close to be usable - at least that’s what ONT told me half a year ago.

Thanks for your suggestion! We have edited as suggested.

2. Mention the genome size limitation in the abstract when you say how it compares to BWA-mem.

It is technically possible to analyze larger genomes, just that it will likely be impractical to use a smartphone for several hours for one analysis. This is too nuanced to explain with a strict word limit for the abstract but we have edited the abstract to clarify our tests were in viral and bacterial genomes:

We benchmark iGenomics using a variety of real and simulated Nanopore sequencing datasets of viral and bacterial genomes and show that iGenomics has performance comparable to the popular BWA-MEM/Samtools/IGV suite, without needing a laptop or server cluster.

Background

3. Mention why they are limited in their reach here: "However, these advances have been limited in their reach, because they are not readily accessible by most individual laboratories and citizen scientists"

We have expanded this point with the following:

Most substantially, the most widely used alignment and analysis tools are not targeting citizen scientists and require expert knowledge on using the command line to install several software packages, run the tools, and understand a variety of file formats.

4. Change to: "Within the past few years, Oxford Nanopore Technologies (ONT, Oxford, UK) has introduced a small inexpensive hand-held sequencing instrument that has made it possible to perform genomics experiments with minimal facilities and in essentially any environment." There is only one working mobile platform at the moment.

Thanks for the suggestion. We have revised as suggested.

5. Add references to: "Nanopore sequencing technology works by measuring the change in ionic current as a DNA molecule is passed through a nanopore (REF). The DNA molecules are typically a few hundred to tens of thousands of nucleotides long (REF), ~~sampled from random positions throughout the genome.~~" The last part of the sentence only applies to "shot-gun" sequencing, so I would delete it. I would rather add a sentence on the maximum lengths. Also, I would expand this description a bit.

Thank you for the suggestion. We have revised as "Nanopore sequencing technology works by measuring the change in ionic current as a DNA molecule is passed through a nanopore (Goodwin et al. 2016). The DNA molecules are typically a few hundred to tens of thousands of nucleotides long and the longest reported read has exceeded 2 million nucleotides (Payne et al. 2019)."

6. Change to: “which are typically stored in fastq format and saved for further processing, especially read alignment and variant analysis.” Some applications use fast5 directly or fasta (with the drawback of losing quality information).

Edited as suggested.

7. Rephrase: “The standard approach for analyzing reads is to align the reads to a reference genome on high-end laptops, servers, or even supercomputers. While this is possible for those with access to these technologies, these requirements are out of reach for many researchers and citizen scientists.” I see your point, but pretty much everyone that has access to a smart phone and a MinION also has access to a laptop! Please, rephrase.

I (Schatz) routinely use my iPhone for diverse applications that could instead be performed on my laptop: web browsing, email, twitter & other social media platforms, reading ebooks and papers, photography, online shopping, and many other tasks. However, I prefer my iPhone for many of these tasks because the user experience is more convenient and the portability allows me to use my iPhone in scenarios that would be awkward with a larger laptop. We expect the main uses of iGenomics will be similar since it is easy to share results, perform analysis on the go, and many other uses even though these could also be performed on a laptop or server.

We have rephrased: The standard approach for analyzing reads is to align the reads to a reference genome on high-end laptops, servers, or even supercomputers. While this is possible for those with access to these technologies, these requirements may be out of reach for many researchers and citizen scientists. Instead, iGenomics just requires the sequenced reads, which can be loaded from the phone itself, the internet, or else where, and can allow anyone to perform sequence analysis and mutation identification. As with other mobile applications (web browsing, email, social media, etc), iGenomics can be used in a variety of settings that would be awkward to perform with a larger laptop, and many users will also prefer the more intuitive user interface. Furthermore, there are many important scenarios where analyzing these data without high-end computing hardware is desirable, especially in remote environments. Interestingly, current iOS devices, including both iPads and iPhones, have significant computing resources, with clock speeds and onboard RAM approaching that of high-end laptop computers. That said, no standalone genomics analysis software is currently available for iOS devices.

8. Rephrase and change: “Consequently, iGenomics is leading the shift of DNA analysis software and sequencing tools towards mobile devices and marks a great leap forward towards widespread DNA analysis by non-bioinformatically trained doctors, researchers and citizen scientists.” I think this is a bit presumptuous. iGenomics is a very exciting tool and definitely a step in the right direction, but it has yet to show that people will use it and it is still not as fast and versatile as simple laptop-based tools. Furthermore, to be used by medical doctors it needs to be validated for medical purposes and accredited by institutions such as the

FDA - which is far from being realized. So, please use caution is this sentence, and rephrase it accordingly.

Thanks for the suggestion. Note for small genomes, iGenomics has performance comparable to laptop-based tools. See Figure 2 for runtime comparison and Figure 3 for accuracy comparison. We have rephrased as: "Consequently, iGenomics is leading the shift of DNA analysis software and sequencing tools towards mobile devices and marks a great leap forward towards widespread DNA analysis by non-bioinformatician students, researchers and citizen scientists."

Also, it still needs a laptop or a sever for basecalling, so it's not really an option yet. As far as I can see it can't interact with the MinIT yet either, or?

Correct, the basecalling currently needs a server or laptop. But there are many collaborative scenarios where iGenomics can be useful especially with the Dropbox and other import mechanisms as well as providing students and citizen scientists an easy to learn interface for the analysis. We have made this explicit in the discussion:

As the MinION uses a USB connection that is not available on an iPhone or iPad, users will first need to collect the raw sequencing data on their laptop or server as well as use these platforms to base call the signal data into nucleotide sequences.

Results

9. It's not clear what the sentence means: "iGenomics brings a high level of interaction to DNA sequence analysis" Interaction is what way? How is that different to GUI-based analysis on a laptop?

As we describe, users can browse the alignment data in an easy-to-use and intuitive manner, using the same pinch-zooming, pan scrolling, and tap for more information gestures they know from browsing the internet or using popular apps like Facebook. This allows for iGenomics to be used with almost no learning curve.

10. Why doesn't it offer a function to load data from the phone's internal or external SDs? I think that would be very helpful.

iGenomics actually does this support this -- If you go to the "files" app on your iPhone and have any fasta or fastq files there, you can open those in iGenomics. We have rephrased this for clarity: iGenomics provides multiple options for inputting both reads and reference files: selecting from a variety of default files for common bacterial genomes, using Dropbox to choose a file, or loading a fasta or fastq file straight into iGenomics from another app such as Google Drive, Files, or Airdrop.

11. This option is only interesting for a very limited user base. Are you planing to extend it to more pathogens? “For example, iGenomics comes with a preloaded known mutations file that indicates certain mutations in the influenza genome, which, if present, cause resistance to certain antivirals (Hussain et al. 2017) .”

We are considering adding additional mutation lists. Fortunately, this is a very straightforward process: Adding new known mutations files to iGenomics is as simple as creating a new tab-delimited file similar to a BED file listing the mutations and loading the file into iGenomics using one of the described methods.

12. Change to: “Another ~~powerful~~ view within the analysis pane is the coverage profile” Otherwise, it reads more like an advertisement.

Corrected.

13. Change to: “The Summary window, accessible from within the analysis pane, has four pages and provides some useful tools for more detailed analyses.” It is not clear what high-level analysis means.

Edited as “The Summary window, accessible from within the analysis pane, has four pages and provides some useful tools for a high-level overview of the data.”

14. Figure 1: Did you test that the color selection is distinguishable for red-green colorblind people?

We have revised all of the figures to use a more colorblind friendly palette. Specifically, we are now using the high contrast Tol_bright palette, which is color-blind friendly according to https://thenode.biologists.com/data-visualization-with-flying-colors/research/

15. Add details: “and the read characteristics would mirror reads produced by real-world sequencers.” How was this achieved?

Here we mean the read length and overall error rates were chosen to be representative of the sequencing platforms. We have expanded this discussion with: Accordingly, reads of length 100bp and sequence error rate of 1.0% were simulated to mirror reads generated by Illumina sequencers and reads of length 1,000bp and sequence error rate of 10.0% were simulated to mirror reads generated by Oxford Nanopore sequencers. Sequencing errors were introduced at random to mimic the errors produced by sequencers.

16. Figure 2: Please, increase the font size of the legend.

We have updated the legend as requested.

17. Figure 2: “bwa for the BWA/Samtools pipeline.”

Here it reads like that was run on an iPhone, too. But later you say “For context, the BWA-MEM/ Samtools runtime for these data sets was computed on an early 2015 MacBook Pro with a 2.9GHz Intel Core i5 running OS X El Capitan while the iGenomics runtime was computed on a 2017 iPhone 8 with a 2.39 GHz A11 Bionic Chip running iOS 12.3.1.” Please change that to make the comparison more transparent! Also, how did you choose this laptop? Please, add. Otherwise, it makes the runtime comparison a bit arbitrary.

This was the hardware that we had available. iPhones use a specialized processor, and so it is not possible to exactly match the hardware between the iPhone and laptop. Our goal was to show the runtime behavior was similar between the iPhone and laptop although the specifics will of course depend on the CPUs, RAM available, and other technical specifications of the devices used. We have revised the caption as:

***Figure 2**: Runtimes for simulated reads from five reference genomes. The data sets consisted of reads averaging 100x coverage and a reference file. Each data set was tested, defined as aligning then variant calling, using iGenomics running on an iPhone and a BWA/Samtools pipeline running on a laptop. The technical specifications of the iPhone and laptop used for testing are described in the Results section. Each trend line indicates the runtime for each data set using the denoted alignment and analysis software- iG for iGenomics and bwa for the BWA/Samtools pipeline. The dotted lines indicate the specific measurements recorded.*

18. “as all of these important viruses can be analyzed in under 5 seconds on a mobile device.” - this is very impressive!

Thank you!

19. Figure 3: The legend font is so small that it’s very very hard to read. Please increase it substantially.

We have updated the legend as requested.

20. Figure 4: It is not clear what the lines refer to? Why aren’t those dots? Are those different genome sizes or what? How did you get the 4-5x faster if the x-axis ranges from 10 - 60s for BWA runs and the y from 1.8 to 2.4s for iGenomics? This figure is a bit confusing without much more information added on how to read it.

*We have revised the figure to make this more clear. We have also clarified the legend as follows: **Figure 4:** iGenomics runtime vs. BWA/Samtools pipeline runtime for simulated datasets of constant mutation rates and sequence error rates of H1N1 for varying read lengths.*

21. This is hard to argue since the two pipelines were run on two totally different systems. Please, rephrase. “In all of the cases examined, iGenomics had a faster runtime than the

desktop alignment pipeline of BWA-MEM/Samtools (Figure 5). This is likely due to a difference in how iGenomics and the desktop software store the alignments in memory."

We believe this statement to be correct. We have measured the runtime for each of the steps in the BWA-MEM/Samtools pipeline and found the alignment time is not the dominant phase of the analysis but rather the file format conversions and scanning for variants. In contrast, within iGenomics we use internal data structures for tracking the alignments that do not require any of this overhead. Also see Reviewer 2 comment 1 below.

22. Figure 5 This should be a table. Also it needs more details on how to interpret it. Are any of these viruses sequenced with Sanger? It might be good for the readers to show a % similarity to the true genome sequence here - which is much more intuitive, at least on a quick glance, to most readers.

We have revised the table as suggested. None of these samples were sequenced with Sanger, although for Zika there is a consensus genome that we use for comparison using the whole genome alignment algorithm nucmer (part of MUMmer). We have revised this as: "For Zika, the test was based on using a ground-truth set of mutations derived by comparing the consensus genome with nucmer (Kurtz et al. 2004) to the isolate Zika virus/H.sapiens-tc/KHM/2010/FSS13025 (GenBank: KU955593.1) as the reference."

23. Influenza typing I don't think such a detailed introduction to Influenza is needed. Why is this a separate section and not part of the "Viral Genome Analysis" one? Also, what is the advantage of the pan-genome approach? Please, add a bit more information why you performed this test and what it shows the potential users.

Many of our tests were focused on demonstrating the performance and accuracy of iGenomics on Influenza genomes, as well as its practical use-cases to identify known mutations so we believe this level of detail was appropriate to motivate the following analysis. Importantly, our pan-genome allowed us to identify the specific type of flu in the sample as well as identify any mutations present. We have clarified the pan-genome analysis as follows:

For this, we developed an influenza "pan-genome reference sequence" containing representatives for three different Influenza genomes <...> The identity of the A segment is identified by evaluating which of the potential segment types has the largest number of alignments. In the context of iGenomics, the pan-genome approach is preferable to aligning the reads against multiple Influenza genomes in isolation because it is much simpler and allows for typing and variant identification at the same time. Worth noting, the pan-genome approach does not sacrifice accuracy or performance, as shown below.

Discussion

24. It required million dollars to carry out the sequencing, not “large million dollar instruments”. Change accordingly.

Edited as “DNA sequencing has advanced tremendously over the past three decades; a process that once required hundreds of millions of dollars can now be done on handheld devices costing only $1,000 (Shendure et al. 2017).”

25. Change: “For high-end servers and laptops, this software already exists; for mobile devices, iGenomics is the first comprehensive solution for researchers and citizen scientists ~~to easily analyze sequence data using a device that they already own~~.” Many people own a laptop.

Edited as “For high-end servers and laptops, this software already exists; for mobile devices, iGenomics is the first comprehensive solution for researchers and citizen scientists to easily analyze sequence data.”

26. Again, this is not really true. I carry out MinION sequencing in many places around the world: with my laptop. “Unlike traditional DNA mapping software, iGenomics can be used in virtually any location because of the inherent portability of mobile devices like the iPad and iPhone.” Please, rephrase.

See our response to comment 7 above. We have rephrased as: iGenomics can be used in virtually any location because of the inherent portability of mobile devices like the iPad and iPhone.

27. I would delete this sentence: “Interestingly, while Objective-C is sometimes an afterthought for computationally intensive apps, iGenomics leverages the language’s capabilities to generate both a unique user experience and fast analysis times.” or explain why it is usually not used.

We have deleted this sentence.

28. Why talk about corona here, when you do not mention it in the introduction or show any comparisons for it in the paper?

Our expertise is primarily in flu genome analysis, but we highlighted these results to demonstrate a timely and important potential application for iGenomics. We also now mention the COVID19 sequencing in the introduction with several citations.

29. This is a crucial part that has not been discussed in the paper at all: “For example, by using Airdrop to both import and export data from iGenomics, a researcher can analyze DNA in remote locations without any internet connectivity.

“Why would I analyze my data on an iPhone if I “Airdrop” the data to the phone from my laptop? Why not simply run it on the laptop in the first place? You should really discuss this. Are there any cases or will there be any applications where you won’t need a laptop/server to basecall the reads? E.g. will iGenomics work with the MinIT? I really think this is an interesting step in the right direction, but since basecalling will need to be performed on a laptop/server anyway, I think the use for a phone-based software is limited. Please, discuss this issue and possible future solutions.

We have expanded the discussion on this point:

“For example, by using Airdrop to both import and export data from iGenomics, a researcher can analyze DNA in remote locations without any internet connectivity. As the MinION uses a USB connection that is not available on an iPhone or iPad, users will first need to collect the raw sequencing data on their laptop or server as well as use these platforms to base call the signal data into nucleotide sequences. However, once sequencers are available that can read DNA directly into iOS devices, iGenomics will work out of the box to allow for importing of this sequenced data, eliminating the requirement for a laptop in the end-to-end analysis pipeline.”

30. Again: “In fact, Oxford Nanopore has announced that they hope to have a new sequencer, named the “SmidgION”, that connects directly to iOS devices available for researchers within the next year.” They have been saying that for many years. Also, this statement really needs a reference if you want to leave it in!

We admit this has been delayed by several years but we include this as it points to how this technology may evolve in the future. We have revised as:

In fact, Oxford Nanopore has announced that they hope to have a new sequencer, named the “SmidgION”, that connects directly to iOS devices available for researchers in the near future (https://nanoporetech.com/products/smidgion).

31. This is only true if the basecalling can be done on the mobile device, a topic you haven’t touched on in this current draft. Please, do so.

See comment 29 above.

Methods

32. As this paper is partly targeted towards a citizen-science audience, please try to word this a bit easier to understand: “The Burrows-Wheeler Transform (BWT) is constructed by lexicographically sorting the cyclic permutations of the input genome appended by a end-of-string character.” Most non-computer scientists will have no idea what that means.

We have rephrased as: The Burrows-Wheeler Transform (BWT) is constructed by lexicographically sorting the cyclic permutations of the input genome appended by a end-of-string character. By convention, we use a dollar sign (‘$’) as the end-of-string character,

which has a lexicographical value less than any letter in the English alphabet and ensures the end of the original sequence can be found. For example, the cyclic permutations of the string "CAT" with the end-of-string character "$" are: "CAT$", "AT$C", "T$CA", and "$CAT", which can be sorted as "$CAT", "AT$C", CAT$" and "T$CA". This sorted list creates what is known as the Burrows-Wheeler Matrix (BWM). Then, to compute the BWT from the sorted permutations, the last character of each row in the matrix is extracted in order and appended to a string.

33. please change to "The main challenge with the General User Interface (GUI) was to create one"

Edited as "Graphical User Interface (GUI)"

34. Change to "The solution to developing this ~~unique~~ interactive analysis screen"

Edited.

~~~~~~

Reviewer 2

1. Why BWA-MEM/Samtools tools (algorithms) were chosen for the work (not minimap2, for example)?

We selected them because these tools are extremely widely used in genomics. They also use the most similar data structures and alignment techniques to iGenomics so that we could focus on a comparison of the hardware capabilities rather than the use of minimizers or other algorithmic differences. We also note the alignment stage of the analysis only represents a fraction of the total runtime so that end-to-end runtime is similar between BWA and Minimap2. Specifically, we measured the runtimes for simulated H1N1 reads with read length 100bp, sequence error rate of 0.01 (1%) and mutation rate of 0.1 (10%) using BWA and Minimap2:

	BWA	***Minimap2***
Index	*0.010 s*	*0.011 s*
Align	*0.889 s*	*0.429 s*
Total	*0.899 s*	*0.440 s*

However, after the alignments are computed, several steps remain using samtools to convert and sort the alignments and then identify variants (the time is essentially identical using either set of alignments):
~~~~~~

1. *SAM to BAM: 0.24s*
2. *BAM to sorted BAM: 0.24s*
3. *BAM to BCF: 2.62s*
4. *BCF to VCF: 0.01s*
5. *Total = 3.11s*

While minimap2 is clearly faster, the point still stands that the majority of time is spent on variant identification. Addressing this point, we added the following text to the manuscript:

To further explore the performance of iGenomics, we also compared the BWA+SAMtools pipeline described above with that of Minimap2 (Li 2018) + SAMtools, using exact same steps in SAMtools after the SAM file was generated by the respective alignment tool. For the simulated H1N1 reads with read length 100bp, sequence error rate of 0.01 (1%) and mutation rate of 0.1 (10%), we found that the indexing and alignment time was insignificant compared to the amount of time spent on variant calling: the alignment time for BWA was 0.899s (22.42% of the total runtime), 0.440s for Minimap2 (12.39% of the total runtime), and 3.11s for identifying variants by converting the SAM file to BAM (0.24 s), sorting the BAM file (0.24 s), identifying candidate variants in BCF format (2.62 s), and computing the final variant calls (0.01 s). Thus, while Minimap2 is noticeably faster than BWA, the majority of time is spent on variant calling.

2. Page 2. "Due to the lower amount of processing power in mobile devices compared to high-end desktop computers or servers, iGenomics is limited in the size of the genome that can be processed" I think it would be useful to specify what is the limit (in terms of genome size and coverage) for the application?

Please see our response to reviewer 1 comment 2 above.

3. Page 6 mentions "the BWA-MEM/Samtools runtime for these data sets was computed on an early 2015 MacBook Pro with a 2.9GHz Intel Core i5 running OS X El Capitan while the iGenomics runtime was computed on a 2017 iPhone 8 with a 2.39 GHz A11 Bionic Chip running iOS 12.3.1." Were the same devices used in the other tests of the work? Was iGenomics tested on other iOS devices?

Yes for the timing and accuracy measurements, this same device was used everywhere in the paper. However, we have tested iGenomics on other iPhones and iPads with alternate screen dimensions to ensure usability. We have emphasized this point in the results section with this new text:

All timing results presented in this paper use these hardware configurations, although we tested iGenomics on several iPhone and iPad models to ensure usability across screen sizes and system resources.

4. Page 8-9 Section "Viral Genome Analysis". Figure 5. It is not completely clear to me what was considered as a "ground truth" in each comparison (what is 'nucmer'?). Additional comments would be helpful here.

We have remade this and all of the figures and tables for clarity. Nucmer is a widely used tool for whole genome alignment (it is a component of MUMmer) that allows us to directly align the sample genome to the reference genome to identify variants. This also allowed us to construct an independent ground truth as well as compare the variant calls between iGenomics and BWA/Samtools. We have add this text to clarify this point:

For Zika, the test was based on using a ground-truth set of mutations derived by comparing the consensus genome with nucmer (Kurtz et al. 2004) to the isolate Zika virus/H.sapiens-tc/KHM/2010/FSS13025 (GenBank: KU955593.1) as the reference.

5. Section "Simulated read accuracy analysis" Why the simulations were stopped at the error rate 0.2 ("sequence error rates of 0.01, 0.1, and 0.2")? An error rate of nanopore reads is generally higher (in the other sections the value 10.0 was used).

We believe the values may have been misinterpreted here. For this section the error rate is reported as the fraction of error and not the percentage of error so that "0.2" indicates 20% sequencing error. Indeed this error rate exceeds the current average error rate of about 10%. See (Wick et al, 2019, Genome Biology) for a benchmarking of current neural network based basecalling methods. To clarify this point, we added the following sentence:

Note that an error rate of 0.2 represents a 20% error rate, and exceeds the current average error rate for Nanopore sequencing (Wick et al. 2019).

6. Section "Simulated read runtime analysis" The sizes of reference genomes are not shown in the text, only in Figure 1, where they are hard to find.

We added the genome sizes to the text where the genomes are introduced:

(1) phiX174, a widely used control sequence for Illumina sequencing (Genbank:NC_001422.1, 5386bp);
(2) a Zika virus genome (isolate Zika virus/H.sapiens-tc/KHM/2010/FSS13025, 10807bp);
(3) a H3N2 influenza genome (A/California/7/2004(H3N2), 13382bp);
(4) a H1N1 influenza genome (A/New York/205/2001(H1N1), 13568bp); and
(5) an Ebola genome (isolate Ebola virus/H.sapiens-wt/SLE/2014/Makona-G3686.1, 18957bp).

7. Figure 10, 6-th column ("G") - Is "3" in the correct row?

We have reworded the caption to clarify the point of this view:

Figure 10: A table showing how the coverage profile is represented within iGenomics, summarizing how the reads align to the reference genome (an example of reads aligned to a reference genome is shown in Figure 1). As can be seen in the 6th column, there is a mutation where the base C was found when the reference was base G.

~~~~~~

Reviewer 3

SUMMARY
The authors present iGenomics, a comprehensive genome analysis iOS application that enables read alignment, variant calling, and an easy visualization of results. It was developed in Objective-C using the FM-index, banded dynamic programming and, according to the authors, other high-performance bioinformatics techniques. The app has been benchmarked against real and simulated sequencing datasets, and the results show that its performance is comparable to other desktop tools (i.e. BWA-MEM, samtools, IGV).

HIGHLIGHTS
- The paper is very well written and it is very pleasant to read.
- The app runs the complete analysis on the iOS device.
- Data can be loaded directly from Dropbox or imported via any iOS app capable of sharing files.
- Results can be shared using Dropbox, AirDrop, Google Drive, Mail, etc.
- The performance of the app was benchmarked using simulated and non-simulated data, obtaining very good results in comparison to desktop tools.
- The app is very easy to use for experts and non-experts (citizen scientists).

Thank you for your support!

MAJOR REMARKS
My main concern is regarding the incompatibility of this app with Android devices. There are more than 2 billion Android devices around the planet. I suggest the authors, in a future version, using Ionic [1], Xamarin [2], Xojo [3] or any other cross-platform framework to develop the app for different systems and architectures. Besides, if you move to Android, you can use external APIs and/or Docker containers in order to increase functionality in a fast and efficient way. Finally, and with the advent of 5G, I guess that calling an API in order to get all the
~~~~~~

functionality seems the easiest and fastest solution, rather than coding every single algorithm back again in Objective-C (or Swift, see point 8 below).

Thank you for your suggestions. We will certainly keep this in mind for future releases although this is beyond the scope of what we can accomplish now.

MINOR REMARKS

1. I suggest changing the title to "iGenomics: Comprehensive DNA Sequence Analysis on a mobile device" since the app runs also on an iPad, not only on a smartphone.

Thank you for the suggestion. We discussed this at length internally and decided to keep the original title.

2. Page 11: line 12: there is a cite (Chan et al. 2020) that is not listed in the references.

Thanks for pointing this out, we have fixed the omission.

3. Page 11, lines -8 and -9: change "extract" for "obtain" in "Then, to extract the BWT…", since you have "is extracted" in the following line as well.

We have edited this for clarity.

4. Page 11: regarding BWT sorting, isn't there any recent advance on this field better than using Quicksort in O(n log n)? (see [4])

Yes, there are faster algorithms available, including linear time algorithms (e.g. (Belazzougui et al. 2020). However, given that iGenomics is targeted towards small genomes, index construction consumes a negligible amount of the runtime and is amortized over all of the reads aligned. We have rephrased this as:

iGenomics uses a version of QuickSort, a divide-and-conquer sorting algorithm, because on average it takes O(n log n) time for n objects to be sorted. Although there are now some more efficient BWT construction algorithms (Belazzougui et al. 2020), given iGenomics is targeted towards relatively small genomes (<100,000bp), the amount of time for BWT sorting is negligible compared to the time to align the reads.

5. Page 13, line 6: why a match of a 20bp substring of the read?

This value is just used for finding exact seeds, and is similar to the default seed length used in other tools. We have clarified as:

To determine where to begin the band computation, iGenomics attempts to exact match a 20bp substring of the read. A substring length of 20bp was chosen as we found that represented the optimal tradeoff in terms of performance and reliability of identifying alignments.

6. Regarding the iOS code, have you used any Objective-C design patterns aside from the traditional MVC? If not, I suggest taking a look to [5], [6] and [7]. Using design patterns eases building and maintaining apps, along with the chance to add new functionalities in a fast way.

For the GUI, we use inheritance and subclassing, such as with the AlignmentGridView and CoverageGridView being subclasses of QuickGridView. Additionally, the alignment logic is encapsulated to ensure independence from the rest of the codebase and the variant identification logic is also encapsulated for the same reason.

7. Why Objective-C and not Swift? I have my own opinion, but I want to hear your reasons.

Objective-C makes it very easy to interact with C primitives in the same/similar way as C; Swift is a bit different to interact with these primitives

8. The app's Github is a bit cluttered (doc/ppt/image files mixed with code files). I suggest arranging the repository for any prospective user.

We have cleaned up the GitHub so that it's clear what is code and what are the resources used to validate our results

9. The tutorial on the web [8] does not match my experience on the iPhone. For example, "5. Select the parameters for mapping the reads" is different from the screen I got in that step.

We have updated the screenshots to show the latest version.

10. Is the data and software available in the public domain under a Creative Commons license?

Our license is shown in the README in our GitHub repo. Briefly: Permission is hereby granted, free of charge, to any person obtaining a copy of this software and associated documentation files (the "Software"), to deal in the Software without restriction, including without limitation the rights to use, copy, modify, merge, publish, distribute, sublicense, and/or sell copies of the Software.

Improvements

11. Build a cross-platform app, in particular, for Android devices, and develop using design patterns.

The focus of this paper was to indicate the advancement in allowing for alignment and intuitive analysis of sequences on a mobile device. In the next iteration of iGenomics, a cross-platform framework can be used to ensure that both the Android and iOS populations have the ability to align and analyze DNA on their devices. We agree with these suggestions, as well as the APIs, for future work. As of now API access is limited to regions where there is internet access, and there are still large portions of the world with no internet access where iGenomics can be used. There are some global satellite-based internet solutions, such as Starlink, designed to provide internet coverage everywhere, however these are still in development and would not yet allow an API-based approach to iGenomics to be used anywhere.

12. Regarding the reference genome, maybe it will be very useful in the near future to move to a graph genome structures [9], rather than working with the actual linear reference.

As you point out in [9] (Schatz & Cosgrove, 2019, Genome Biology, Graph genomes article collection), we are well aware of these developments, although this is beyond the scope of what we can accomplish now. We will keep this in mind for future versions of the software.

CONCLUSION

Finally, and with the advent of 5G, I guess that calling an API in order to get all the functionality seems the easiest and fastest solution, rather than coding every single algorithm back again in Objective-C (or Swift, see point 8 below).

This is a possibility to offload some computation to the cloud, although 5G it is currently not very widespread and iGenomics is designed to work in any environment, including without internet access at all. Furthermore, our paper demonstrates that the hardware is very capable for onboard processing.

Thus, the paper can be accepted when the minor comments are completed.

Thank you for your suggestions. We have addressed all of your minor comments.

Marcos Colebrook, Ph.D.
Associate Professor
Depto. Ingeniería Informática y de Sistemas
Universidad de La Laguna
