# Supplementary material for: iGenomics: Comprehensive DNA sequence analysis on your Smartphone
Source: Gigascience. 2020 Dec 7;9(12):giaa138. doi: 10.1093/gigascience/giaa138 (PMC7720420; doi:10.1093/gigascience/giaa138)
Supplement: giaa138_Response_to_Reviewer_Comments_Original_Submission [file giaa138_response_to_reviewer_comments_original_submission.pdf]

Author's Response To Reviewer Comments

Close

Please see the response letter PDF file.

Close
