# Supplementary material for: iGenomics: Comprehensive DNA sequence analysis on your Smartphone
Source: Gigascience. 2020 Dec 7;9(12):giaa138. doi: 10.1093/gigascience/giaa138 (PMC7720420; doi:10.1093/gigascience/giaa138)
Supplement: giaa138_Reviewer_2_Report_Original_Submission — Yulia Suvorova -- 5/12/2020 Reviewed [file giaa138_reviewer_2_report_original_submission.pdf]

Reviewer Report

Title: iGenomics: Comprehensive DNA Sequence Analysis on your Smartphone

Version: Original Submission **Date:** 5/12/2020

Reviewer name: Yulia Suvorova

Reviewer Comments to Author:

Dear Authors,
I think that iGenomics is an exciting application that will allow many researchers and doctors to quickly analyze genomic sequences outside their labs. I believe that it would be especially useful in the current pandemic situation.
I have several questions and comments about the work described below.
With kind regards,
Yulia Suvorova

1. Why BWA-MEM/Samtools tools (algorithms) were chosen for the work (not minimap2, for example)?
2. Page 2. "Due to the lower amount of processing power in mobile devices compared to high-end desktop computers or servers, iGenomics is limited in the size of the genome that can be processed" I think it would be useful to specify what is the limit (in terms of genome size and coverage) for the application?
3. Page 6 mentions "the BWA-MEM/Samtools runtime for these data sets was computed on an early 2015 MacBook Pro with a 2.9GHz Intel Core i5 running OS X El Capitan while the iGenomics runtime was computed on a 2017 iPhone 8 with a 2.39 GHz A11 Bionic Chip running iOS 12.3.1." Were the same devices used in the other tests of the work? Was iGenomics tested on other iOS devices?
4. Page 8-9 Section "Viral Genome Analysis". Figure 5. It is not completely clear to me what was considered as a "ground truth" in each comparison (what is 'nucmer'?). Additional comments would be helpful here.
5. Section "Simulated read accuracy analysis" Why the simulations were stopped at the error rate 0.2 ("sequence error rates of 0.01, 0.1, and 0.2")? An error rate of nanopore reads is generally higher (in the other sections the value 10.0 was used).
6. Section "Simulated read runtime analysis" The sizes of reference genomes are not shown in the text, only in Figure 1, where they are hard to find.
7. Figure 10, 6-th column ("G") - Is "3" in the correct row?

Methods

Are the methods appropriate to the aims of the study, are they well described, and are necessary controls included? Choose an item.

Conclusions

Are the conclusions adequately supported by the data shown? Choose an item.

Reporting Standards

Does the manuscript adhere to the journal's guidelines on [minimum standards of reporting?] Choose an item.

Choose an item.

Statistics

Are you able to assess all statistics in the manuscript, including the appropriateness of statistical tests used? Choose an item.

Quality of Written English

Please indicate the quality of language in the manuscript: Choose an item.

Declaration of Competing Interests

Please complete a declaration of competing interests, considering the following questions:

- Have you in the past five years received reimbursements, fees, funding, or salary from an organisation that may in any way gain or lose financially from the publication of this manuscript, either now or in the future?
- Do you hold any stocks or shares in an organisation that may in any way gain or lose financially from the publication of this manuscript, either now or in the future?
- Do you hold or are you currently applying for any patents relating to the content of the manuscript?
- Have you received reimbursements, fees, funding, or salary from an organization that holds or has applied for patents relating to the content of the manuscript?
- Do you have any other financial competing interests?
- Do you have any non-financial competing interests in relation to this paper?

If you can answer no to all of the above, write 'I declare that I have no competing interests' below. If your reply is yes to any, please give details below.

I declare that I have no competing interests

I agree to the open peer review policy of the journal. I understand that my name will be included on my report to the authors and, if the manuscript is accepted for publication, my named report including any attachments I upload will be posted on the website along with the authors' responses. I agree for my report to be made available under an Open Access Creative Commons CC-BY license (http://creativecommons.org/licenses/by/4.0/). I understand that any comments which I do not wish to be included in my named report can be included as confidential comments to the editors, which will not be published.

Choose an item.

To further support our reviewers, we have joined with Publons, where you can gain additional credit to further highlight your hard work (see: https://publons.com/journal/530/gigascience). On publication of this paper, your review will be automatically added to Publons, you can then choose whether or not to claim your Publons credit. I understand this statement.

Yes Choose an item.
