# Supplementary material for: iGenomics: Comprehensive DNA sequence analysis on your Smartphone
Source: Gigascience. 2020 Dec 7;9(12):giaa138. doi: 10.1093/gigascience/giaa138 (PMC7720420; doi:10.1093/gigascience/giaa138)
Supplement: giaa138_Reviewer_3_Report_Original_Submission — Marcos Colebrook -- 5/13/2020 Reviewed [file giaa138_reviewer_3_report_original_submission.pdf]

Reviewer Report

Title: iGenomics: Comprehensive DNA Sequence Analysis on your Smartphone

Version: Original Submission **Date:** 5/13/2020

Reviewer name: Marcos Colebrook

Reviewer Comments to Author:

REPORT ON THE PAPER
"iGenomics: Comprehensive DNA Sequence Analysis on your Smartphone"
by Aspyn Palatnick, Bin Zhou, Elodie Ghedin, Michael C. Schatz

SUMMARY

The authors present iGenomics, a comprehensive genome analysis iOS application that enables read alignment, variant calling, and an easy visualization of results. It was developed in Objective-C using the FM-index, banded dynamic programming and, according to the authors, other high-performance bioinformatics techniques. The app has been benchmarked against real and simulated sequencing datasets, and the results show that its performance is comparable to other desktop tools (i.e. BWA-MEM, samtools, IGV).

HIGHLIGHTS

- The paper is very well written and it is very pleasant to read.
- The app runs the complete analysis on the iOS device.
- Data can be loaded directly from Dropbox or imported via any iOS app capable of sharing files.
- Results can be shared using Dropbox, AirDrop, Google Drive, Mail, etc.
- The performance of the app was benchmarked using simulated and non-simulated data, obtaining very good results in comparison to desktop tools.
- The app is very easy to use for experts and non-experts (citizen scientists).

MAJOR REMARKS

My main concern is regarding the incompatibility of this app with Android devices. There are more than 2 billion Android devices around the planet. I suggest the authors, in a future version, using Ionic [1], Xamarin [2], Xojo [3] or any other cross-platform framework to develop the app for different systems and architectures. Besides, if you move to Android, you can use external APIs and/or Docker containers in order to increase functionality in a fast and efficient way.

Finally, and with the advent of 5G, I guess that calling an API in order to get all the functionality seems the easiest and fastest solution, rather than coding every single algorithm back again in Objective-C (or Swift, see point 8 below).

MINOR REMARKS

1. I suggest changing the title to "iGenomics: Comprehensive DNA Sequence Analysis on a mobile device" since the app runs also on an iPad, not only on a smartphone.
2. Page 11: line 12: there is a cite (Chan et al. 2020) that is not listed in the references.
3. Page 11, lines -8 and -9: change "extract" for "obtain" in "Then, to extract the BWT…", since you have "is extracted" in the following line as well.

4. Page 11: regarding BWT sorting, isn't there any recent advance on this field better than using Quicksort in O(n log n)? (see [4])
5. Page 13, line 6: why a match of a 20bp substring of the read?
6. Regarding the iOS code, have you used any Objective-C design patterns aside from the traditional MVC? If not, I suggest taking a look to [5], [6] and [7]. Using design patterns eases building and maintaining apps, along with the chance to add new functionalities in a fast way.
7. Why Objective-C and not Swift? I have my own opinion, but I want to hear your reasons.
8. The app's Github is a bit cluttered (doc/ppt/image files mixed with code files). I suggest arranging the repository for any prospective user.
9. The tutorial on the web [8] does not match my experience on the iPhone. For example, "5. Select the parameters for mapping the reads" is different from the screen I got in that step.
10. Is the data and software available in the public domain under a Creative Commons license?

IMPROVEMENTS

11. Build a cross-platform app, in particular, for Android devices, and develop using design patterns.
12. Regarding the reference genome, maybe it will be very useful in a near future to move to a graph genome structures [9], rather than working with the actual linear reference.

CONCLUSION

Although I am really aware that the authors cannot fulfill the major requirements or improvements in the next version of the revised paper, I believe that writing some lines in this direction will help the prospective readers.

Thus, the paper can be accepted when the minor comments are completed.

Marcos Colebrook, Ph.D.
Associate Professor
Depto. IngenierÃ-a InformÃ¡tica y de Sistemas
Universidad de La Laguna

REFERENCES

[1] https://ionicframework.com/
[2] https://dotnet.microsoft.com/apps/xamarin
[3] https://www.xojo.com/
[4] Okanohara D., Sadakane K. (2009) A Linear-Time Burrows-Wheeler Transform Using Induced Sorting. In: Karlgren J., Tarhio J., HyyrÃ¶ H. (eds) String Processing and Information Retrieval. SPIRE 2009. Lecture Notes in Computer Science, vol 5721. Springer, Berlin, Heidelberg.
[5] Chung, C. (2011), Pro Objective-C Design Patterns for iOS, Apress.
[6] https://developer.apple.com/documentation/swift/migrating_your_objective-c_code_to_swift
[7] https://developer.apple.com/library/archive/documentation/Cocoa/Conceptual/CocoaFundamentals/CocoaDesignPatterns/CocoaDesignPatterns.html
[8] http://schatz-lab.org/iGenomics/tutorial/
[9] Schatz, M.C., Cosgrove, A. Graph genomes article collection. Genome Biol 20, 25 (2019). https://doi.org/10.1186/s13059-019-1636-0.

Methods

Are the methods appropriate to the aims of the study, are they well described, and are necessary controls included? Choose an item.

Conclusions

Are the conclusions adequately supported by the data shown? Choose an item.

Reporting Standards

Does the manuscript adhere to the journal's guidelines on minimum standards of reporting? Choose an item.

Choose an item.

Statistics

Are you able to assess all statistics in the manuscript, including the appropriateness of statistical tests used? Choose an item.

Quality of Written English

Please indicate the quality of language in the manuscript: Choose an item.

Declaration of Competing Interests

Please complete a declaration of competing interests, considering the following questions:

- Have you in the past five years received reimbursements, fees, funding, or salary from an organisation that may in any way gain or lose financially from the publication of this manuscript, either now or in the future?
- Do you hold any stocks or shares in an organisation that may in any way gain or lose financially from the publication of this manuscript, either now or in the future?
- Do you hold or are you currently applying for any patents relating to the content of the manuscript?
- Have you received reimbursements, fees, funding, or salary from an organization that holds or has applied for patents relating to the content of the manuscript?
- Do you have any other financial competing interests?
- Do you have any non-financial competing interests in relation to this paper?

If you can answer no to all of the above, write 'I declare that I have no competing interests' below. If your reply is yes to any, please give details below.

I declare that I have no competing interests

I agree to the open peer review policy of the journal. I understand that my name will be included on my report to the authors and, if the manuscript is accepted for publication, my named report including any attachments I upload will be posted on the website along with the authors' responses. I agree for my report to be made available under an Open Access Creative Commons CC-BY license (http://creativecommons.org/licenses/by/4.0/). I understand that any comments which I do not wish to be included in my named report can be included as confidential comments to the editors, which will not be published.

Choose an item.

To further support our reviewers, we have joined with Publons, where you can gain additional credit to further highlight your hard work (see: https://publons.com/journal/530/gigascience). On publication of this paper, your review will be automatically added to Publons, you can then choose whether or not to claim your Publons credit. I understand this statement.

Yes Choose an item.
